# The Impact of Non-Fiscal Mandatory and Voluntary Policies and Interventions on the Reformulation of Food and Beverage Products: A Systematic Review

**DOI:** 10.3390/nu16203484

**Published:** 2024-10-14

**Authors:** Jessica Packer, Semina Michalopoulou, Joana Cruz, Disha Dhar, Claire Stansfield, Helena Kaczmarska, Russell M. Viner, Oliver Mytton, Simon J. Russell

**Affiliations:** 1 Population, Policy and Practice Research and Teaching Department, UCL Great Ormond Street Institute of Child Health, University College London, London WC1N 1EH, UK; s.michalopoulou@ucl.ac.uk (S.M.); joana.cruz@ucl.ac.uk (J.C.); disha.dhar@ucl.ac.uk (D.D.); helena.kaczmarska.22@alumni.ucl.ac.uk (H.K.); r.viner@ucl.ac.uk (R.M.V.); o.mytton@ucl.ac.uk (O.M.); s.russell@ucl.ac.uk (S.J.R.); 2 EPPI-Centre, UCL Social Research Institute, University College London, London WC1H 0NR, UK; c.stansfield@ucl.ac.uk

**Keywords:** food policy, nutrition policy, nutritional labelling, nutrition, food label

## Abstract

**Background/Objectives**: Low quality diets are a risk factor for non-communicable diseases; therefore, improving diet quality is a public health and policy priority in the UK and elsewhere. Reformulating food/beverage products to make them healthier may be an effective approach. Evidence suggests that fiscal interventions, notably taxes/levies on soft drinks, can lead to reformulation but the evidence for voluntary or mandated non-fiscal interventions is less clear. We aimed to review and synthesise contemporary evidence to determine whether non-fiscal policies/interventions result in the reformulation of food/beverage products **Methods**: In April 2023, we systematically searched ten international academic and nine grey literature databases. We included real-world study designs, all nutrients, in- and out-of-home sectors, and studies published from 2013, to ensure policy relevancy. We excluded modelling studies. Using the Synthesis Without Meta-Analysis method we conducted vote counting of studies based on the direction of effect and narrative synthesis by intervention type. Risk of bias was assessed using a tool developed by the EPPI-Centre and quality was assessed using GRADE. **Results**: We included 77 real-world studies from 19 countries, reporting 100 non-fiscal policies/interventions. Most commonly, these were reduction targets (*n* = 44), front-of-pack labels (*n* = 23), and advertising standards (*n* = 9). Most interventions were voluntary (*n* = 67), compared to mandatory (*n* = 33), and focused on the in-home sector (*n* = 63). The vote counting results showed non-fiscal policies/interventions overall led to improvements in reformulation in 60/63 studies with a valid direction of effect (95%, 95% CI 0.869, 0.984, *p* < 0.001). Mandatory implementations were more successful than voluntary implementations with 15/15 showing an improvement (100%, 95% CI 0.796, 1], *p* < 0.001), compared 40/43 showing an improvement (93%, 95% CI 0.814, 0.976, *p* < 0.001). Most of the studies were of low quality, due to the observational nature of the studies. Sodium was the most commonly targeted nutrient (*n* = 56) and was found to be reformulated in most studies. Causation is difficult to establish from real-world studies, but evidence suggests that regulatory and multi-component strategies may be effective at driving reformulation. **Conclusions:** Non-fiscal policies/interventions can play an important role in driving reformulation, alongside fiscal measures. This work was funded by the National Institute for Health and Care Research PRP-PRU-02-15-Healthy Weight and registered on Open Science Framework.

## 1. Introduction

Non-communicable diseases account for 74% of deaths globally, with unhealthy diets being a key modifiable risk factor [1,2]. In the UK, the average diet exceeds the government recommendations for calories, sugar, saturated fat, and salt intake, and does not meet the recommendations for fruit, vegetables or fibre intake [3,4,5]; a pattern observed globally [6]. This is partly driven by pre-packaged ultra-processed foods (UPFs) consumption, which is high and increasing globally (e.g., an average daily intake of 60% in the UK, 58% in the US, 42% in Australia and 30% in Mexico); and has been linked to harmful health effects [7,8,9,10].

There is strong evidence linking low quality diet and health problems, including high sugar and saturated fat intake, and low fibre, fruit and vegetable intake with an increased risk of cardiovascular diseases (CVD), hypertension, and cancers [11,12,13,14,15,16]. Evidence suggests that bringing UK diets in line with the government recommendations, could avoid 33,000 deaths per year [17].

To improve the healthiness of average population diets, many governments have introduced mandatory and voluntary food policies, including fiscal and non-fiscal interventions [18,19]. A key component of improving diets in many countries is improving the nutritional quality of products via reformulation, which includes the reduction of salt, sugar and fat, or the addition of nutrients such as fibre and protein [20]. As an upstream policy action, the reformulation of products has been shown to be an effective mechanism to improve population diets [21]. Since reformulation does not rely on individual agency or choice, it may be more effective and equitable compared to downstream interventions [22,23].

Fiscal policies or interventions may encourage industry to reformulate their products, including revenue raising measures (taxes) or expenditure measures (subsidies), such as the UK Soft Drinks Industry Levy (SDIL) [24]. The SDIL was shown to be effective at increasing the reformulation of soft drinks [25] but fiscal policies can be challenging and expensive to implement, with public and industry pushback [26]. Non-fiscal policies or interventions can be mandatory or voluntary and include front of pack nutrition labels or claims, back of pack nutritional information, menu labels, nutrient reduction targets, nutrient limits, advertising regulations, industry or retailer self-imposed standards; and they can be less challenging to implement, especially voluntary policies which do not require legislation. Often these policies aim both to influence consumer behaviours and encourage the food and beverage industry to reformulate their products [21]. There is an evidence-based pathway of how food reformulation impacts child and adolescent obesity [27]. This pathway is supported by evidence from experimental studies suggesting reformulation of calorie content in products can reduce total energy intake, leading to weight loss, with minimal compensation effects [28].

There is a wealth of evidence to suggest that reformulated products are widely accepted by consumers, and lead to improvements in the nutritional composition of products purchased and consumed by consumers [21]. There is one systematic review that assessed the impact of regulatory interventions relating to trans-fatty acids specifically [29]. However, there have been no reviews to date examining the impacts of non-fiscal policies and interventions on the reformulation of all nutrients This study aims to systematically review the evidence assessing the impact of any non-fiscal policy or intervention, for any food and beverage product, in both sectors (in-home e.g., products sold in supermarkets or out-of-home e.g., products sold in restaurants), on the reformulation of any nutrient (including calories, sugar, salt, fat, protein, fibre, vitamins and products healthfulness).

## 2. Materials and Methods

This systematic review was conducted using EPPI-Reviewer Version 6 [30], reported in accordance with the PRISMA checklist [31], and registered with Open Science Framework [32].

### 2.1. Eligibility Criteria and Search Strategy

We included any real-life quasi-experimental study designs such as pre-post studies, repeated cross-sectional studies and difference in differences, and other quasi-experimental designs. We included articles published from 2013 onwards, across all countries and languages, to ensure policy relevance considering significant changes in the global food system, regulatory landscape, and advances in food processing technologies. The intervention eligibility criteria included any study that examined the exposure of a non-fiscal voluntary or regulatory policy, with any outcome related to the reformulation of products, including all nutrients (including salt, sugar, saturated fat, trans-fatty acids and micronutrients) and both in- and out-of-home sectors. Outcome measures included any measurement of product reformulation and change in nutrient content (grams, milligrams, millilitres, kilojoules, kilocalories, percentage of total product). We defined appropriate comparators, i.e., comparisons to product formulations before policies were introduced or unaffected markets (if other criteria are met). Both peer-reviewed and non-peer-reviewed grey literature (including government reports, dissertations/theses and conference presentations) were eligible for inclusion, to ensure that all relevant evaluations were captured. Exclusion criteria were any studies examining only fiscal policies, the reformulation of alcohol products (due to the difference in policymaking relating to these products), modelling studies, reviews, commentaries, editorials, conference abstracts (due to lack of detail, not lack of peer-review), case studies, books and or opinion pieces. The grey literature records were assessed using the same criteria as the academic database records, with requirements for the detail presented, especially regarding the methodology and the results. See Table S1 in the supplementary file for the full inclusion/exclusion criteria.

The search strategy was developed in collaboration with an information scientist at the EPPI-Centre (CS). Systematic searches of the following resources covering business, healthcare, psychology, public policy, science and social science were conducted: Econlit (EBSCO); Embase (OVID); CINAHL (EBSCO); Cochrane Library CENTRAL; Medline (OVID); ProQuest Central: ASSIA, ABI Inform Global and PAIS; PsycINFO (OVID); Web of Science: Social Science, Emerging Sources and Science citation indexes. Grey literature searches were conducted using the following resources: World Cancer Research Fund (WCRF) NOURISHING Database, WHO institutional repository, World Obesity Federation, NCD Alliance, OSF preprint, Google, BASE (Bielefeld Academic Search Engine), CORDIS European Commission and Policy Commons. The database searches were conducted in English and used a variety of synonyms and terms (including MESH terms) to capture bibliographic records that contained the following three concepts: (i) reformulation or recipe development; (ii) foods or food composition; and (iii) interventions, policies, standards. See Table S2 in the supplementary file for the full search strategy for each database and further details about the search and Table S3 for a step-by-step guide to conducting/replicating the review.

Database searches were conducted on 24 April 2023 and duplicates were removed in Endnote Version 20 [33] and EPPI-Reviewer Version 6 [30]. EPPI-Reviewer Version 6 was also used for the screening and to manage the review, but no other functions were used. In addition, a ‘cited by’ search of related systematic reviews and key articles that became known to the review team during the planning stages of the review was conducted using Google Scholar, see Table S2 in the supplementary file for further details.

Records were screened in double by combinations of two reviewers from a team of four reviewers (JP, SM, JC, HK) on both title and abstract and then full-text using the inclusion criteria. Discrepancies at either stage were resolved through consensus.

### 2.2. Quality Assessment

To assess the risk of bias of the articles we used a tool developed by the EPPI-Centre at the UCL Institute of Education [34]. It includes a critique of the data sampling, data collection, measures, analysis and inferences made and was originally used in a systematic review of responses to standardised packaging of cigarettes and rolling tobacco [35]. Bias assessments were conducted independently by two reviewers (DD, AG) and any discrepancies were jointly resolved. Quality assessments of the included studies were conducted using GRADE (Grading of Recommendations, Assessment, Development, and Evaluations) by two reviewers (JP, SJR).

### 2.3. Data Extraction

Data extracted were study characteristics (primary author, publication year, country, study design), intervention or policy characteristics (regulation type, intervention details, nutrient targeted, product types), key results (nutrient reformulation and degree of change).

### 2.4. Data Synthesis

Meta-analysis of the studies was not possible due to the heterogeneity of the study designs, the nutrients targeted and the outcome measures. The synthesis was conducted using vote counting based on the direction of effect by implementation type, following the Synthesis Without Meta-Analysis (SWiM) reporting guidelines [36]. Outcomes were classified into four outcome categories according to the direction of effect. The categories were improvement (where the nutrients within the studied products were reformulated in the intended direction); mixed effect (where nutrients within the studied products were reformulated in both the intended and the unintended direction); no effect (where there was no observed change in the nutrients within the studied products); and worsening (where the nutrients within the studied products were reformulated in the unintended direction). For the vote counting, only the studies that showed a direction of effect were included, creating a binary metric. The confidence interval, *p*-values and harvest plot were calculated or created following the methods outlined in the Cochrane Handbook [36,37,38]. We narratively synthesised findings by intervention type, nutrient (including degree of change), product category (type of food or beverage), country of policy implementation, sector (in- vs. out-of-home), and time period to achieve reformulation [39]. We adopted groupings for intervention type from a related study: labelling requirements; use of nutrition and health claims; front of pack labels or use of health logos; governmental measures to enforce companies to reformulate [40].

## 3. Results

### 3.1. Study Selection

**Figure 1:**
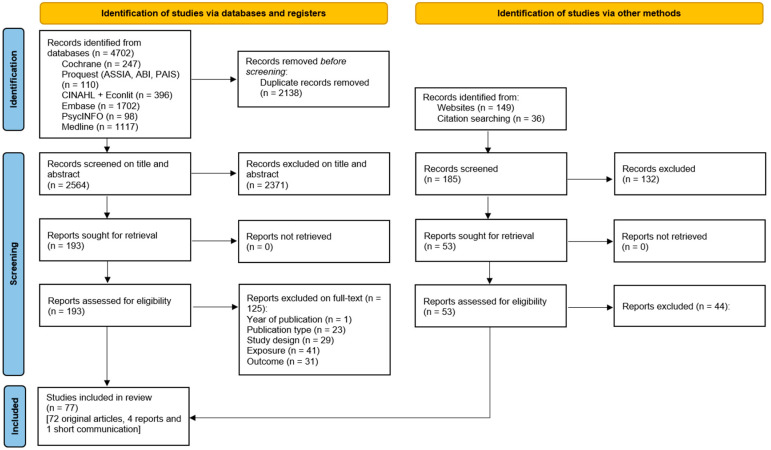
Flowchart of the screening process.

From database searching, 4702 articles were identified, with 2564 remaining after duplicate removal, see Figure 1 for the flowchart. Following title and abstract screening, 2371 were excluded and 193 articles were screened on full-text, with an additional 53 from grey literature searches and citation searching, of which 77 articles were final includes (72 original articles, four grey literature articles, one short communication).

### 3.2. Study Descriptions

See Table 1 for a descriptive summary of the included studies and Table S4 for additional descriptive detail. 

From the 77 included studies, a total of 100 interventions were assessed. The studies were conducted in Europe (*n* = 25: Austria = 1, Belgium = 1, Denmark = 1, France = 4, The Netherlands = 3, Ireland = 1, UK = 9, Spain = 2, Slovenia = 2), United States (US) (*n* = 12), Canada (*n* = 4), South America (*n* = 10: Brazil = 2, Chile = 6, Colombia = 1, Peru = 1), Australia (*n* = 13), New Zealand (*n* = 4), South Africa (*n* = 1), South Korea (*n* = 1), or across multiple countries (*n* = 5: Australia and New Zealand = 1, 22 EU countries = 1; Global = 1; Latin America = 1, UK and Latin America = 1, UK and China = 1, US and France = 1, US and Canada = 1). Nearly 90% of the interventions studied were conducted in high-income countries. The most common quasi-experimental study designs of the included studies were repeated cross-sectional (*n* = 50), pre-post (*n* = 14), cross-sectional (*n* = 5), longitudinal observational (*n* = 5), difference in differences (*n* = 2), and one study used both pre-post and repeated cross-sectional methods.

The policy implementations were predominantly voluntary (*n* = 67), compared to mandatory (*n* = 33). The main nutrients targeted were nutrients to avoid including sodium (*n* = 56), sugar (*n* = 38), energy (*n* = 30), saturated fat (*n* = 29), trans-fatty acids (*n* = 13) and fats (*n* = 11); also, nutrients to encourage including fibre (*n* = 13), protein (*n* = 11), non-nutritive sweeteners (*n* = 5), and vitamins/micronutrients (*n* = 2).

Most of the studies examined policies that were focused on the in-home sector (*n* = 63), compared to out-of-home (*n* = 8), or studies that examined both (*n* = 6). The majority of the assessed products were packaged foods (*n* = 48), mix of packaged foods and beverages (*n* = 16), only beverages (*n* = 3), and fast-food products (*n* = 10).

Policies and interventions included voluntary reduction targets (*n* = 44), front of pack labels or claims (*n* = 23), advertising standards (*n* = 8, included voluntary pledges from industry or Chilean regulations), back of pack nutrition information (*n* = 7), mandatory nutrient limits (*n* = 4, i.e., upper content limits for trans-fatty acids), prohibition of cooking methods (*n* = 2), menu labelling (*n* = 7), industry-led reformulation strategy (*n* = 4), school standards (*n* = 1). The reduction targets were further categorised as set by the government (*n* = 32), industry (*n* = 6), non-governmental organisations (*n* = 2), World Health Organisation (*n* = 1), or a combination (*n* = 2). See Table 2 for the most common examples of each policy and intervention from the included studies.

### 3.3. Vote Counting Results

Overall, non-fiscal policies and interventions were found to have an effect on intended reformulation of nutrients, with 63 studies having a valid direction of effect and 60 showing an improvement (95%, 95% CI 0.869, 0.984, *p* < 0.001). See Figure S1 in the supplementary file for the harvest plots. An estimate of the proportion of effects favouring reformulation following non-fiscal policies or interventions was calculated by considering implementation of the interventions. For the articles assessing mandatory implementation there were 17 total, 15 with a valid direction of effect of which all showed an improvement in the intended nutrient reformulation (100%, 95% CI 0.796, 1, *p* < 0.001); two showed no change. For the articles assessing voluntary implementations there were 51 total, 43 with a valid direction of effect of which 40 showed an improvement in the intended nutrient reformulation and three showed a worsening (93%, 95% CI 0.814, 0.976, *p* < 0.001); five showed a mixed effect and three showed no change. There were seven articles assessing mixed implementation, of which five had a valid direction of effect and all showed an improvement in the intended nutrient reformulation (100%, 95% CI 0.566, 1, *p* = 0.062). Regarding the quality of the studies, all 15 of the mandatory implementation studies and the seven with mixed implementation were assessed as low quality. For the voluntary implementation studies, two were assessed as very low quality, two as moderate and the rest as low quality. Sensitivity analysis excluding the two studies assessed as having very low quality showed no impact on the findings and is presented in the supplementary file, Table S5.

### 3.4. Narrative Synthesis

The reformulation outcomes by intervention or policy type, and implementation are presented descriptively in Table 3. Of the most common interventions, the majority of the government-set reduction targets (22/26, 85%), multi-pronged interventions (14/18, 78%), front of pack label or health claims (10/11, 91%) and menu labelling interventions (3/5, 60%) led to improvements in the nutritional quality of products. Of the less common interventions, one-of-four industry-led reduction targets (25%) and two-of-three industry/retailer-led strategies (67%), did not lead to reformulation i.e., improvements in the nutritional quality of products. The remaining seven intervention types led to improvements in nutritional quality through product reformulation, including mandatory or voluntary nutrient declarations, non-governmental organisations or World Health Organisation-led reduction targets, school standards, mandatory limits, and advertising standards. We considered reformulation outcomes by mandatory or voluntary implementation and found a higher proportion of mandatory interventions led to improvements (89%), compared to voluntary (79%); however, fewer studies assessed voluntary interventions (17 compared to 53 mandatory). We did not find any mandatory interventions that led to adverse outcomes, compared to eight voluntary interventions (16%) that showed worsening or mixed results. Only two of the mandatory policies studied led to no change and they both evaluated menu labelling at fast-food chain restaurants (either a salt shaker image to indicate a product was high in sodium in chain restaurants or energy content on menu boards) [53,55].

We considered reformulation outcomes by sector, nutrient and implementation type, as presented in Table 4. In terms of sector, we found that in-home interventions (exclusively or mixed) led more frequently to reformulation (82%), compared to out-of-home (67%). Sodium was the most studied and reformulated nutrient leading to improvements in the overall content (42/56, 75%). Trans-fatty acids were studied less frequently but had a high rate of being reduced or removed from products via reformulation (9/13, 69%). Other nutrients, sugar, energy and saturated fat were studied across a similar number of studies (ranging from 38–29%, respectively) and led to reformulation in similar proportions (59–50%, respectively).

By product type, most of the included studies assessed a range of mixed packaged food and most led to reformulations that improved the nutritional content of products (78%). For studies that assessed specific product types, breakfast cereals, condiments/sauces, and yoghurt were found to be reformulated with an improved nutritional content in all included studies (100%); and in one study chocolate was found to be reformulated, leading to a worsening of nutritional quality. Results were more varied for fast-food, ready meals and beverages than the other categories.

The scope and scale of the policies and interventions studied were often broad and applied to most products within a sector. Policies applied to all products across a sector rather than a narrower subset, were more common for mandatory policies and for the in-home sector/packaged foods. For example, all products that had trans-fatty acid content were required to state that on their packaging, according to the US regulation that was reported in two studies [45,110]; and the Chilean front of pack label policy applied to all products that crossed the threshold for salt, sugar, saturated fat and energy that were studied in six studies [39,41,50,51,56,74]. Comparatively, out-of-home sector policies were often only applied to fast-food restaurants or restaurant chains. For example, mandatory menu-labelling was a common policy across five studies, applying to restaurants with more than 20 locations. This represented few restaurants in scope but a large number of products (over 35,000 menu items) [46,53,54,55,115]. Some studies assessed single or few products covered by the policy or intervention. For example, De Kock et al., 2016 focused on stock cubes but the South African mandatory salt reduction targets covered bread, breakfast cereals, spreads, savoury snacks, potato chips, processed meat, sausages, soup powder, gravy powder, savoury powder and stock cubes [44].

Table 5 summarises study results in terms of improvements or worsening of nutritional quality and the direction of change. We found that 62 studies showed an improvement in the nutritional quality of the studied products (81%), with the majority reporting a decrease in unhealthy nutrients. Three studies found a worsening in nutritional quality, due to increases in unhealthy nutrients (4%); six studies showed no changes (8%); and six studies showed mixed changes across the nutrients included (8%).

We were unable to present the results on the time period to achieve reformulation due to the study designs and the lack of information reported in the studies.

### 3.5. Bias and Quality Assessment

The bias assessment of the studies is presented in Table S6. Most of the studies were assessed as having low concerns (*n* = 53, 69%), followed by some or minor concerns (*n* = 20, 26%), and then those with high concerns (*n* = 4, 5%). Of the studies that were judged to be of high concern, this was either due to insufficient information on the study methods (*n* = 2), or conflicts of interest due to funding by industry (*n* = 2). The studies of high concern did not affect overall findings. The quality assessment of the studies showed that most were low quality (*n* = 73, 94%), compared to moderate (*n* = 2, 3%) and very low (*n* = 2, 3%). The low-quality assessment of the studies was predominantly due to the study designs being uncontrolled. Therefore, issues with confounding remain, meaning that the reformulation results cannot be causally linked to the studied policies. The two studies that used difference in differences designs were both found to be of low concern (*n* = 2 for controlled study designs).

## 4. Discussion

In this systematic review, we included 77 studies assessing non-fiscal reformulation policies or interventions. We found that non-fiscal measures can lead to reformulation and improvements in the nutritional composition of food and drink products. Voluntary interventions are more commonly implemented and comprised the majority of evaluated interventions; however, mandatory interventions led to higher rates of product reformulation and improvements in nutritional quality. Studies assessing multi-pronged interventions, for example trans-fat nutrient declaration, content limits and cooking prohibition, were also found to lead to higher rates of reformulation. Interventions that targeted or led to reductions sodium were evaluated more than other nutrients and the majority of the interventions focused on the in-home/retail sector. While we cannot draw strong conclusions about causality given the real-world nature of included studies, this review provides evidence that non-fiscal interventions can be effective at improving the nutritional quality of food and beverage products.

Our findings support previous research showing that non-fiscal policies and interventions can be effective at driving product reformulation [117,118]. In contrast to previous studies, we assessed the impact of a range of non-fiscal interventions and across many macro-nutrients and were able to extend findings beyond previous reviews that focused on fiscal policies, front-of-pack labels interventions and trans-fatty acids [29,117,118]. Consistent with past work, our findings suggest that mandatory policies rather than voluntary are more likely to be effective (i.e., lead to changes in the nutrient composition of foods) [119].

Sodium was the most commonly targeted nutrient, and led to the highest rates of reformulation, compared to the other nutrients (i.e., the reduction of sodium from products) [117]. There has been a history of salt reformulation starting in the UK in 1991 [120,121], with other countries subsequently adopting sodium reduction programmes (75 countries as of 2015, up from 32 in 2010) [122]. While studies show that sodium is often reduced in products, this often falls short of targeted levels (for example, in the UK only 70% of sauces met 2017 targets) [97]. The voluntary nature of sodium targets, in addition to technical challenges relating to palatability may limit the achievable reduction. In contrast, reformulation of trans-fatty acids was often achieved by mandatory policies, e.g., mandatory labelling of trans-fatty acid content or regulations on cooking techniques. Each nutrient presents different challenges and needs around reformulation (e.g., consumer awareness, salt as a preservative vs. trans-fatty acids as a by-product of production) and may require different approaches. Sodium and trans-fatty acids may be easier to reduce from products compared to nutrients such as sugar and sugar has not been actively targeted through policy measures for as long as sodium and trans-fatty acids [117,123]. Sugar may be harder to reformulate because it impacts on taste, may contribute to the bulk or weight of a product and is cheap [20,124].

The impact that reformulation can have on consumer behaviours and health outcomes was beyond the scope of this review and warrants further research. The mechanisms and impact on health also varies by nutrient. However, there is some evidence linking non-fiscal policies that drove reformulation to improvements in population dietary intakes and health. For example, mandatory trans-fat labelling regulations in Canada, led to decreases in the recorded levels of trans-fatty acids present in breast milk [125]; government-set sodium reduction targets in South Korea were linked to reductions population blood pressure and hypertension prevalence [86]; and industry-led sodium reduction pledges in the US and government targets in the UK were associated with decreases in salt intake [126,127]. A modelling study from New Zealand also projected a mandatory front of pack labelling scheme would lead to reductions in mortality [128].

Structural interventions or population-level policies can be more equitable than interventions that rely on individual agency [129,130]. For this reason, reformulation policies might be expected to be more equitable, although the equity impacts were often not considered in the included studies. Different policies may have varying impacts on equity. For example, mandatory front of pack labelling could affect demographic groups differently if manufacturers assume that these groups are more or less likely to engage with labels. This perception could then influence manufacturing decisions about which products to reformulate (i.e., focusing on products popular with higher demographic groups).

Focusing on individual change has been shown to have limited effects and increasingly there is acknowledgment among academic, public health professionals and policy makers that there is a need to consider and adjust the underlying food system [23,131]. There is evidence that non-fiscal interventions may be more effective at driving reformulation when part of a broader programme of policies, that includes fiscal and other measures. Countries with the greatest reformulation success have tended to implement a range of multi-pronged and mandatory policies. Six of the included studies were conducted in Chile, and assessed the impact of the mandatory 2016 regulation for front of pack warning labels and advertising and sales restrictions (applicable to products that crossed specified thresholds for sodium, sugar, fats and calories) [132]. All of the included studies that assessed the collective impact of policies post-implementation, found that reformulation was achieved in terms of reductions in sugar, calories, sodium and saturated fat content [39,41,50,51,56,74]. This was a coordinated government policy, spanning multiple interventions, with a roll-out phased over 36 months. There was minimal industry involvement during the initial regulation, which was viewed as an integral component for success by academic experts [133]. The food and beverage industry is known to pushback on policies and interventions aimed at driving reformulation [134,135]. These tactics have been categorised by the WCRF as delaying (e.g., arguing for longer consultation periods, more research); dividing (e.g., voluntary rather than mandatory); deflecting (e.g., reframing the issues or claiming that self-regulation is effective); and denying (e.g., citing a lack of evidence and discrediting effectiveness) [134]. The industry tactic of offering self-regulation or voluntary policies, as opposed to mandatory regulation, is likely to be effective in avoiding product reformulation. Our findings are supportive of this suggestion, showing that voluntary policies were less effective at driving reformulation. The three studies from our review that showed a worsening in the nutritional quality of the products were all voluntary interventions [70,92,96]. It is likely that mandatory regulations are required to initiate equal change across the food sector and create ‘a level playing field’ [136]. An important policy consideration will be around the impact of any policies and interventions on the affordability of products, as the accessibility and affordability of food is a known key issue [137].

The strengths of this study include the comprehensive search strategy and the broad inclusion criteria relating to the intervention type and nutrient. We found many relevant studies and the majority were deemed to have low bias, but to be of low quality. Of the grey literature records that we included, all four were government reports and found through the BASE database or the WCRF NOURISHING database [43,48,61,72]. An example of a grey literature record that we did not include was a report by the Food and Drink Federation, which did not provide enough detail to be included [138]. There are inherent limitations with real-world studies, particularly with the predominant study designs being pre-post and repeated cross-sectional. They cannot be randomised and are often not controlled or isolated (only two studies were controlled), so is it not possible to determine causality or the singular effect of an intervention. But real-world studies do have a validity that experimental studies do not. Due to the heterogeneity of the studies (including the intervention types, countries, nutrient types, product types), we did not undertake meta-analyses. Instead, we performed the SWiM method and a narrative synthesis with descriptive data. The limitations of using the vote-counting method are that it does not provide any information on the magnitude of effects, nor does it account for differences in the relative sizes of the studies. This was also a limitation of the narrative synthesis, where we gave equal weight to all studies regardless of size, bias or quality. We were also unable to present findings on the time required to achieve reformulation. To ensure policy relevancy, we excluded studies published before 2013, meaning that earlier research is not summarised here.

## 5. Conclusions

This review provides evidence that a range of non-fiscal interventions can contribute to improvements in the nutritional quality of food and beverage products. Most of the evidence relates to sodium and the in-home sector, while there is less evidence regarding sugar. Together with previous research, this review suggests that mandatory policies and interventions may be more effective than voluntary measures in driving reformulation. However, studies included in this review were generally of low quality, and we were not ablet to perform meta-analysis due to high heterogeneity. Evidence from real-world studies is valuable but does not account for socioeconomic and cultural factors that may impact food production and supply. Further high-quality evaluations of non-fiscal interventions and policies are required, especially with consideration of individual consumer behavioural and socioeconomic characteristics. Non-fiscal and fiscal policies can dovetail to drive improvements the nutritional quality of food and drink products as part of a programme of measures that seek to address the systemic determinants of obesity.

## Figures and Tables

**Table 1:** High-level descriptive characteristics of the included studies (*n* = 77).

Primary Author, Year, Country	Design	Policy	Sector	Product	Nutrients	Overall Finding (Direction)	Study Quality
Mandatory							
Ale-Chilet (2022), Chile [39]	Pre-post	FOPL—WL; advertising	In + advertising	Cereal	Sugar, Calories, Sodium, SFA	Improvement	Low
Barahona (2022), Chile [41]	Pre-post	FOPL—WL; advertising	In + advertising	Cereal	Sugar, Calories	Improvement	Low
Bates (2020), US [42]	Cross-sectional	FOP—sodium health claims	In	Mixed processed food	Sodium	Improvement	Low
Champion (2020), France [43]	Repeated cross-sectional	BOP nutrition label	In	Cereal	Fat, SFA, Sugars, Sodium, Fibre	Improvement	Low
De Kock (2016), South Africa [44]	Repeated cross-sectional	Mandatory sodium limits	In	Stock cubes	Sodium	Improvement	Low
Garsetti (2016), US [45]	Repeated cross-sectional	BOP trans-fat	In	Spreads	TFA	Improvement	Low
Grummon (2021), US [46]	Pre-post	Menu labelling—calorie	OOH	Fast food	Calorie	Improvement	Low
Jahn (2018), US [47]	Pre-post	School standards	In	Mixed processed food and beverages	Sodium, Energy, Total fat, SFA, Sugar, Fibre, Protein	Improvement	Low
Martinovic (2020), France [48]	Repeated cross-sectional	BOP nutrition label	In	Sauces	Sugar, Calories, Sodium, SFA, protein	Improvement	Low
Monge-Rojas (2017), Latin America [49]	Repeated cross-sectional	BOP TFA, TFA limits, prohibition of cooking method	Both	Mixed processed food	TFA	Improvement	Low
Quintiliano (2020), Chile [50]	Pre-post	FOPL—WL; advertising	In + advertising	Mixed processed food	Sugar, Calories, Sodium, SFA, NNS	Improvement	Low
Reyes (2020), Chile [51]	Repeated cross-sectional + pre-post	FOPL—WL; advertising	In + advertising	Mixed processed food and beverages	Sugar, Calories, Sodium, SFA	Improvement	Low
Saavedra-Garcia (2022), Peru [52]	Pre-post	FOPL—WL	In	Mixed processed food	Sugar, Sodium, SFA, TFA, NNS	Improvement	Low
Sisti (2023), US [53]	Pre-post	Menu warning—sodium	OOH	Fast food	Sodium	No change	Low
Tran (2019), US [54]	Repeated cross-sectional	Menu labelling—calorie	OOH	Fast food	Calorie	Improvement	Low
Wellard-Cole (2018), AUS [55]	Longitudinal observational	Menu labelling—calorie	OOH	Mixed processed food and beverages	Calorie	No change	Low
Zancheta (2021), Chile [56]	Pre-post	FOPL—WL; advertising	In + advertising	Mixed processed food and beverages	Sugar, NNS	Improvement	Low
Voluntary							
Arcand (2014), Canada [57]	Repeated cross-sectional	Government set nutrient reduction targets	Both	Fast food	TFA	Improvement	Low
Bablani (2020), AUS + NZ [58]	Difference-in-differences	FOPL—HSR	In	Mixed processed food	Calories, sugars, SFA, Sodium, Protein, Fibre	Improvement	Moderate
Bandy (2022), UK [59]	Repeated cross-sectional	Government set nutrient reduction targets	In	Mixed processed food	Sodium	Improvement	Low
Bernstein (2020), Canada [60]	Longitudinal observational	BOP sugar labelling (transition period)	In	Mixed processed food and beverages	Calories, Sugars, SFA, Fat, Sodium, Protein, Fibre	Improvement	Low
Brants (2017), The Netherlands [61]	Repeated cross-sectional	Government set nutrient reduction targets	In	Mixed processed food	Sodium, Sugar, SFA	Improvement	Low
Christoforou (2013), AUS [62]	Repeated cross-sectional	NGO led reduction targets	In	Mixed processed food	Sodium	Improvement	Low
Clapp (2018), US [63]	Pre-post	Government set nutrient reduction targets	In	Mixed processed food	Sodium, Calories	Improvement	Low
Curtis (2016), US [64]	Repeated cross-sectional	Government set nutrient reduction targets	In	Mixed processed food	Sodium	Improvement	Low
Eyles (2013), UK [65]	Repeated cross-sectional	Government set nutrient reduction targets	In	Mixed processed food	Sodium	Improvement	Low
Eyles (2018), NZ [66]	Repeated cross-sectional	Government initiated industry pledges; FOPL—HSR	Both	Fast food	Calories, Sodium	Improvement	Low
Fichera (2020), UK [67]	Difference-in-differences	FOPL—MTL	Both	Bread	Sodium	Improvement	Moderate
Garcia (2020), UK, Mexico, Ecuador, and Guatemala [68]	Cross-sectional	Government set nutrient reduction targets	In	Mixed processed food	Calories, Sodium, SFA, Sugar	Improvement	Low
Gressier (2021), UK [69]	Repeated cross-sectional	Government set nutrient reduction targets	In	Cereal + yogurt	Sugar	Improvement	Low
Hashem (2019), UK [70]	Repeated cross-sectional	Government set nutrient reduction targets	In	Chocolate	Sugar	Worsened	Low
He (2014), UK [71]	Repeated cross-sectional	Government set nutrient reduction targets	In	Mixed processed food and beverages	Sodium	Improvement	Low
Health Canada (2018), Canada [72]	Pre-post	Government set nutrient reduction targets	In	Mixed processed food	Sodium	Improvement	Very low
Jensen (2017), Denmark [73]	Longitudinal observational	Retailer led reformulation strategy	In	Mixed processed food	Sodium	Mixed	Low
Kanter (2019), Chile [74]	Pre-post	FOPL—WL; advertising	In	Mixed processed food	Calories	Improvement	Low
Levi (2018), AUS [75]	Repeated cross-sectional	Government set nutrient reduction targets	In + advertising	Mixed processed food and beverages	Calories, Sodium, SFA, Sugar	No change	Low
Lowery (2020), Colombia [76]	Pre-post	Industry advertising and product pledges	In	Soup	Sodium	Improvement	Low
Luger (2018), Austria [77]	Repeated cross-sectional	NGO led reduction targets	Both	Mixed processed food	Total fat, SFA, TFA, Sodium, Sugar, Energy Density, NNS	Improvement	Low
McMenemy (2022), Ireland [78]	Repeated cross-sectional	Industry led reformulation strategy	Both	SSB	Sugar	Improvement	Low
Ni Mhurchu (2017), NZ [79]	Repeated cross-sectional	FOPL—HSR	In	Mixed processed food	Energy, Protein, Total Fat, Carbohydrate, SFA, Sodium, Sugar, Fibre, Vitamin D/B12, Calcium, Iron	Mixed	Low
Moore (2020), UK [80]	Repeated cross-sectional	Government set nutrient reduction targets	In	Mixed processed food and beverages	Energy, SFA, Sugar, Sodium, Protein, Fibre	Improvement	Low
Moran (2022), US [81]	Repeated cross-sectional	Government set nutrient reduction targets	In	Yogurt	Sugar	Improvement	Low
Morrison (2019), AUS [82]	Repeated cross-sectional	FOPL—HSR	In	Mixed processed food	Sodium	Improvement	Low
Nilson (2017), Brazil [83]	Repeated cross-sectional	Government set nutrient reduction targets	In	Mixed processed food	Energy, SFA, Sugar, Sodium, Protein, Fibre	Improvement	Low
Nilson (2017), Brazil [84]	Repeated cross-sectional	Government set nutrient reduction targets	In	Mixed processed food	Sodium	Improvement	Low
Ning (2017), NZ [85]	Repeated cross-sectional	FOPL—Heart Tick	In	Mixed processed food	Sodium	Improvement	Low
Park (2020), South Korea [86]	Repeated cross-sectional	Government set nutrient reduction targets	In	Mixed processed food	Sodium	Improvement	Low
Pérez-Farinós (2016), Spain [87]	Repeated cross-sectional	Government set nutrient reduction targets	Both	Mixed processed food	Sodium	Improvement	Low
Pérez-Farinós (2018), Spain [88]	Repeated cross-sectional	Government initiated industry pledges	In	Mixed processed food	TFA	Improvement	Low
Pinho-Gomes (2023), AUS [89]	Repeated cross-sectional	Industry nutrient reduction pledges	In	Beverages	Sugar, NNS	No change	Low
Pombo-Rodrigues (2017), UK [90]	Repeated cross-sectional	Government set nutrient reduction targets	In	Cereal	Sodium, Sugar	Improvement	Low
Pravst (2017), Slovenia [91]	Repeated cross-sectional	Government set nutrient reduction targets	In	Mixed processed food	Sodium	Mixed	Low
Russell (2021), AUS [92]	Repeated cross-sectional	FOPL—HSR	In	Mixed processed food and beverage	Sugar, NNS	Worsened	Low
Savio (2013), AUS [93]	Pre-post	Government initiated industry pledges; FOPL—Heart Tick	In	Mixed processed food and beverage	Energy, Protein, Total Fat, SFA, Sugar, Fibre, Sodium	Improvement	Low
Sparks (2018), AUS [94]	Repeated cross-sectional	Government initiated industry pledges	In	Processed meat	Sodium	Improvement	Low
Spiteri (2018), France [95]	Repeated cross-sectional	Government set nutrient reduction targets	In	Grocery basket	Sugar, fats, SFA, fibre, sodium	Improvement	Low
Spiteri (2018), AUS [96]	Cross-sectional	Industry nutrient reduction pledges	In	Mixed processed food and beverage	Energy, Protein, SFA, Carbohydrates, Sugar, Fibre, Sodium	Worsened	Low
Tan (2019), UK + China [97]	Repeated cross-sectional	Government set nutrient reduction targets	In	Sauces	Sodium	Improvement	Low
Tassy (2022), Global [98]	Repeated cross-sectional	WHO set nutrient reduction targets	In	Mixed processed food	Sugar, Sodium, SFA, Protein	Improvement	Very Low
Theis (2019), UK [99]	Cross-sectional	Menu labelling	OOH	Fast food	Energy, Fat, SFA, Sugar, Sodium Carbohydrates, Protein	Improvement	Low
Thomson (2016), NZ [100]	Repeated cross-sectional	FOPL—Heart Tick	In	Mixed processed food	Energy, TFA, SFA, Sodium, Calcium, Fibre	Improvement	Low
Trevena (2014), AUS [101]	Repeated cross-sectional	Government set nutrient reduction targets	In	Mixed processed food	Sodium	Improvement	Low
Trevena (2015), AUS [102]	Repeated cross-sectional	Government set nutrient reduction targets	In	Mixed processed food	Sodium	Improvement	Low
Van Dam (2022), France [103]	Cross-sectional	Government set industry commitments AND FOPL—N-S	Both	Processed food and fast food	Overall product healthiness	No change	Low
Van Der Bend (2020), The Netherlands [104]	Repeated cross-sectional	FOPL—Healthy Choices	In	Mixed processed food	Energy, TFA, SFA, Sodium, Fibre, Sugar	Improvement	Low
Vergeer (2022), Canada [105]	Repeated cross-sectional	Industry nutrient reduction pledges	In	Mixed processed food and beverages	Calories, Sodium, SFA, TFA, Sugar	Mixed	Low
Vermote (2020), Belgium [106]	Repeated cross-sectional	FOPL—N-S	In	Cereal	Calories, Sodium, SFA, Fat, Sugar, Fibre, Protein	Improvement	Low
Vlassopoulos (2017), US + France [107]	Repeated cross-sectional	Industry nutrient reduction pledges	In	Mixed processed food and beverages	Calories, Sodium, SFA, Fat, Sugar	Improvement	Very low
Yon (2014), US [108]	Pre-post	Industry led reformulation strategy	In	Milk	Sugar, Calories, Fat, Sugar	Improvement	Low
Zupanič (2019), Slovenia [109]	Repeated cross-sectional	Government initiated industry pledges	In	Mixed processed food and beverage	Sugar	Mixed	Low
Both							
Hooker (2014), US + Canada [110]	Repeated cross-sectional	Government set nutrient reduction targets (voluntary); BOP TFA & Mandatory FOP claims	In	Cookies	TFA	Improvement	Low
Moz-Christofoletti (2021), 22 EU countries [111]	Repeated cross-sectional	Government set nutrient reduction targets; Mandatory ban on added sugars in juice & TFA content	In	Mixed processed food and beverage	Sugar, TFA, Salt, SFA	Mixed	Low
Otite (2013), US [112]	Longitudinal observational	Retailer led reformulation strategy; Mandatory FOP claims	In	Mixed processed food	TFA	Improvement	Low
Temme (2017), The Netherlands [113]	Repeated cross-sectional	Industry nutrient reduction pledges; Mandatory limits on salt in bread	In	Mixed processed food	Sodium	Improvement	Low
Urban (2014), US [114]	Longitudinal observational	Government set nutrient reduction targets; Cooking regulations	OOH	Fast food	Sodium, SFA, TFA	Improvement	Low
Wellard-Cole (2019), AUS [115]	Repeated cross-sectional	Menu calorie labelling; Industry advertising standards	OOH + advertising	Fast food	Sugar, Calories, Sodium, SFA	No change	Low
Zganiacz (2017), AUS [116]	Repeated cross-sectional	BOP nutrition label; FOPL—Heart Tick; NGO reduction targets; Government set reduction targets	In	Mixed processed food	Sodium	Improvement	Low

FOPL, Front of pack label; BOP, back of pack; OOH, out of home; US, United States; UK, United Kingdom; AUS, Australia; NZ, New Zealand; WL, warning label; SFA, saturated fat; TFA, trans-fatty acids; NNS, non-nutritive sweeteners; HSR, Health Star Rating; MTL, Multiple Traffic Light; WHO, World Health Organisation; NGO, non-governmental organization; N-S, Nutri-Score; SSB, sugar sweetened beverages.

**Table 2:** Examples of policies and interventions from the included studies.

Intervention Type	Examples from Included Studies
Reduction targets	UK Sugar Reduction Programme; UK Salt Reduction Targets: Sugar Reduction Pledge by Australian Beverages Council
Front of pack labels or claims	Chilean Warning Label; Australia-New Zealand Health Star Rating; UK Multiple Traffic Light
Advertising standards	Chilean regulation relating to the advertising restrictions for any products classified as high in sugar, salt, saturated fat, or energy; “Responsible self-regulation” agreement to limit advertising and sales of unhealthy beverages in schools
Back of pack nutrition information	Nutrient declarations on products and the inclusion of trans-fatty acid content
Mandatory nutrient limits	Upper limit for trans-fatty acid content
Prohibition of cooking methods	Banning the use of partially hydrogenated oil
Menu labelling	Voluntary menu labelling of nutrients in restaurants
Industry-led reformulation strategies	“Responsible self-regulation” agreement to limit advertising and sales of unhealthy beverages in schools
School standards	Nutrition standards for snacks sold at school

**Table 3:** The proportion of interventions that led to an improvement in the nutritional quality of products, by implementation and intervention type (*n* = 77).

Implementation & Intervention Type (*n* = 77)	Improvement	Worsening	Mixed	No Change
Mandatory (*n* = 17)				
Multi-pronged interventions * (*n* = 6)	6 (100%)	-	-	-
Front of pack labels or health claims (*n* = 2)	2 (100%)	-	-	-
Menu labelling (*n* = 4)	2 (50%)	-	-	2 (50%)
Mandatory nutrient declaration (*n* = 3)	3 (100%)	-	-	-
School standards (*n* = 1)	1 (100%)	-	-	-
Mandatory limits (*n* = 1)	1 (100%)	-	-	-
Overall	15 (88%)	-	-	2 (12%)
Voluntary (*n* = 53)				
Government-set reduction targets (*n* = 27)	22 (81%)	1 (4%)	3 (12%)	1 (4%)
Front of pack labels or health claims (*n* = 9)	7 (78%)	1 (11%)	1 (11%)	-
Industry–set reduction targets (*n* = 4)	1 (25%)	1 (25%)	2 (50%)	-
NGO/WHO reduction targets (*n* = 3)	3 (100%	-	-	-
Industry/retailer-led strategies (*n* = 3)	2 (67%)	-	1 (33%)	-
Advertising standards (*n* = 1)	1 (100%)	-	-	-
Nutrient declaration (*n* = 1)	1 (100%)	-	-	-
Menu labelling (*n* = 1)	1 (100%)	-	-	-
Multi-pronged interventions ** (*n* = 4)	3 (75%)	-	-	1 (25%)
Overall	42 (79%)	3 (6%)	5 (9%)	3 (6%)
Both (*n* = 7)				
Multi-pronged interventions *** (*n* = 7)	5 (71%)	-	1 (14%)	1 (14%)

NGO, non-governmental organization; WHO, World Health Organisation. The multi-pronged interventions for * mandatory = Chilean Warning Label and advertising restrictions; nutrient declaration and content limits and cooking prohibition; ** voluntary = front of pack labels and industry commitments or pledges or Chilean Warning Label and advertising restrictions in the pre-implementation period; *** both = mandatory and voluntary front of pack or nutrient declarations or nutrient content limits and government or industry set reduction targets.

**Table 4:** The proportion of studies achieving each reformulation outcome by sector, nutrient, implementation and product type.

Outcome Category	Improvement	Worsening	Mixed Results	No Change
Sector (*n* = 77)				
In-home (*n* = 56)	46 (82%)	3 (5%)	6 (11%)	1 (2%)
Out-of-home (*n* = 6)	4 (67%)	-	-	2 (33%)
Mixed (*n* = 15)	12 (80%)	-	-	3 (20%)
Nutrient (*n* = 166)				
Sodium (*n* = 56)	42 (75%)	1 (2%)	6 (11%)	7 (13%)
Sugar (*n* = 38)	22 (58%)	3 (8%)	4 (11%)	9 (24%)
Energy (*n* = 30)	15 (50%)	1 (3%)	2 (7%)	12 (40%)
Saturated fat (*n* = 29)	14 (48%)	1 (3%)	3 (10%)	11 (38%)
Trans-fatty acids (*n* = 13)	9 (69%)	-	2 (15%)	2 (15%)
Product type (*n* = 77)				
Mixed packaged * (*n* = 50)	40 (80%)	2 (4%)	6 (12%)	2 (4%)
Breakfast cereals (*n* = 5)	5 (100%)	-	-	-
Fast food (*n* = 5)	3 (60%)	-	-	2 (40%)
Ready meals (*n* = 5)	4 (80%)	-	-	1 (20%)
Condiments/sauces (*n* = 4)	4 (100%)	-	-	-
Beverages (*n* = 3)	2 (67%)	-	-	1 (33%)
Morning goods * (*n* = 3)	3 (100%)	-	-	-
Yoghurts (*n* = 1)	1 (100%)	-	-	-
Chocolate (*n* = 1)	-	1 (100%)		-

* Morning goods includes sweet biscuits, pastries and muffins.

**Table 5:** Summary of the direction of study results.

Study Findings (*n* = 77)	*n*	%	Significant
Improvements in nutritional quality	62	81 (%)	46
Decreases in unhealthy nutrients	57		
Decreases in unhealthy nutrients and increases in healthy nutrients	5		
Worsening in nutritional quality	3	(4%)	2
Increases in unhealthy nutrients	3		
Decreases in healthy nutrients	0		
Studies that observed no-significant changes	6	(8%)	
Studies that observed mixed changes	6	(8%)	

## Data Availability

No new data were created, all is available in the cited papers.

## Supplementary Materials

The following supporting information can be downloaded at: https://www.mdpi.com/article/10.3390/nu16203484/s1, Table S1: PICOS framework inclusion and exclusion criteria; Table S2: Search strategies for each database; Table S3: Step-by-step guide to conducting/replicating this review; Table S4: Full descriptive table of the included studies; Table S5: Sensitivity analysis for the vote counting; Figure S1: Harvest plots summarising the direction of the reformulation influence by implementation of the non-fiscal policies or interventions; Table S6: Overall bias assessment for each of the included studies.
