# Supplementary material for: The Impact of Non-Fiscal Mandatory and Voluntary Policies and Interventions on the Reformulation of Food and Beverage Products: A Systematic Review
Source: Nutrients. 2024 Oct 14;16(20):3484. doi: 10.3390/nu16203484 (PMC11509918; doi:10.3390/nu16203484)
Supplement: Supplementary file 1 [file nutrients-16-03484-s001.zip › nutrients-3183431-supplementary.pdf]

Supplementary file

Table S1. PICOS framework inclusion and exclusion criteria

	Inclusion	Exclusion
**Exposure (intervention)**	Non-fiscal voluntary and regulatory policies, including:<br>• advertising<br>• promotions<br>• out of home calorie labelling	Any fiscal policies e.g., soft drinks industry levy
**Comparison/ study design**	Evaluations, real world studies, including:<br>• Controlled before-and-after studies<br>• Interrupted time series (ITS) studies	Modelling studies
**Outcome measure**	Reformulation of nutrients (e.g., calories, sugar and salt) in food and non-alcoholic drink products, both in and out of home	Alcohol
**Geography**<br>**Languages**<br>**Time**<br>**Publication type**	All<br>All<br>Last 10 years for relevancy<br>Academic or grey literature	None<br>None<br>Papers published before 2013<br>None

Table S2. Search strategies for each database

Medline (Ovid)

1	(polic* or legislation* or pledge* or ban or bans or law or mandatory or legal or regulat* or self-regulat* or quasi-regulation or co-regulat* or government* or guideline* or codes or "industry code" or "code or practice" or compliance or standards).ti,ab. or Public policy/ or Health policy/ or Nutrition policy/ or Food labelling/ or Government regulation/ or Legal epidemiology/	3650962
2	(standard* or strategy or strategies or intervention* or restriction* or voluntary or public private partnership* or initiative* or program* or reformul*).ti,ab.	4914587
3	(label* or advertising or marketing or sales or packag* or serving* or "product development" or national or population* or industry or ((limit* adj3 content) or (portion adj3 size*) or "Unit pricing" or "unit prices" or (Serving* adj3 size*) or (choice adj5 (food* or drink* or beverage* or health*)))).ti,ab.	3472172
4	2 and 3	975728
5	1 or 4	4379650
6	((Food adj product*) or foodstuff* or foods or Nutrition or Meals or beverage* or drink* or nutrition* or nutrient* or calori* or kilocalori* or energy intake or fat* or trans-fat* or trans-fatty acid* or TFA or salt* or sodium or sugar* or "energy density" or "energy dense" or (food adj3 ingredient*) or (food adj3 composition*) or beverage* or Cola or Colas or Soda or sodas or (fizzy adj2 drink*) or (carbonated adj2 drink*) or (soft adj drink*) or "commercial baby food*" or "commercial infant food*" or "infant drink*" or "baby drink*").ti,ab. or exp food/ or exp beverages/ or infant foods/	3541082
7	(reformulat* or "re-formulat*" or (formulat* adj5 (ingredient* or recipe* or processing or manufact* or novel or new)) or (recipe* adj5 (develop* or novel or new or ingredient* or composition or processing or manufact*))).ti,ab.	30422
8	5 and 6 and 7	1522
9	limit 8 to yr="2013 -Current"	1117

PsycINFO (Ovid)

1	(polic* or legislation* or pledge* or ban or bans or law or mandatory or legal or regulat* or self-regulat* or quasi-regulation or co-regulat* or government* or guideline* or codes or "industry code" or "code or practice" or compliance or standards).ti,ab. or Public policy/ or Health policy/ or Nutrition policy/ or Food labelling/ or Government regulation/ or Legal epidemiology/	663544
2	(standard* or strategy or strategies or intervention* or restriction* or voluntary or public private partnership* or initiative* or program* or reformul*).ti,ab.	1306385
3	(label* or advertising or marketing or sales or packag* or serving* or "product development" or national or population* or industry or ((limit* adj3 content) or (portion adj3 size*) or "Unit pricing" or "unit prices" or (Serving* adj3 size*) or (choice adj5 (food* or drink* or beverage* or health*)))).ti,ab.	716819
4	2 and 3	253159
5	1 or 4	849247
6	((Food adj product*) or foodstuff* or foods or Nutrition or Meals or beverage* or drink* or nutrition* or nutrient* or calori* or kilocalori* or energy intake or fat* or trans-fat* or trans-fatty acid* or TFA or salt* or sodium or sugar* or "energy density" or "energy dense" or (food adj3 ingredient*) or (food adj3 composition*) or beverage* or Cola or Colas or Soda or sodas or (fizzy adj2 drink*) or (carbonated adj2 drink*) or (soft adj drink*) or "commercial baby food*" or "commercial infant food*" or "infant drink*" or "baby drink*").ti,ab. or exp food/ or exp "Beverages (Nonalcoholic)"/ or infant foods/	255283
7	(reformulat* or "re-formulat*" or (formulat* adj5 (ingredient* or recipe* or processing or manufact* or novel or new)) or (recipe* adj5 (develop* or novel or new or ingredient* or composition or processing or manufact*))).ti,ab.	6370
8	5 and 6 and 7	146
9	limit 8 to yr="2013 -Current"	98

Embase (Ovid)

1	(polic* or legislation* or pledge* or ban or bans or law or mandatory or legal or regulat* or self-regulat* or quasi-regulation or co-regulat* or government* or guideline* or codes or "industry code" or "code or practice" or compliance or standards).ti,ab. or Public policy/ or Health policy/ or Nutrition policy/ or Food labelling/ or Government regulation/ or Legal epidemiology/	4741527
2	(standard* or strategy or strategies or intervention* or restriction* or voluntary or public private partnership* or initiative* or program* or reformul*).ti,ab.	6601120
3	(label* or advertising or marketing or sales or packag* or serving* or "product development" or national or population* or industry or ((limit* adj3 content) or (portion adj3 size*) or "Unit pricing" or "unit prices" or (Serving* adj3 size*) or (choice adj5 (food* or drink* or beverage* or health*)))).ti,ab.	4617957
4	2 and 3	1404540
5	1 or 4	5782432
6	((Food adj product*) or foodstuff* or foods or Nutrition or Meals or beverage* or drink* or nutrition* or nutrient* or calori* or kilocalori* or energy intake or fat* or trans-fat* or trans-fatty acid* or TFA or salt* or sodium or sugar* or "energy density" or "energy dense" or (food adj3 ingredient*) or (food adj3 composition*) or beverage* or Cola or Colas or Soda or sodas or (fizzy adj2 drink*) or (carbonated adj2 drink*) or (soft adj drink*) or "commercial baby food*" or "commercial infant food*" or "infant drink*" or "baby drink*").ti,ab. or exp food/ or exp beverages/ or infant foods/	3695620
7	(reformulat* or "re-formulat*" or (formulat* adj5 (ingredient* or recipe* or processing or manufact* or novel or new)) or (recipe* adj5 (develop* or novel or new or ingredient* or composition or processing or manufact*))).ti,ab.	39912
8	5 and 6 and 7	2278
9	limit 8 to yr="2013 -Current"	1702

Web of Science- social science + emerging sources + science citation indexes

1	TS=(polic* or legislation* or pledge* or ban or bans or law or mandatory or legal or regulat* or self-regulat* or quasi-regulation or co-regulat* or government* or guideline* or codes or "industry code" or "code or practice" or compliance or standards)	8,134,598
2	TS=(standard* or strategy or strategies or intervention* or restriction* or voluntary or public private partnership* or initiative* or program* or reformul*)	7,852,181
3	TS=(label* or advertising or marketing or sales or packag* or serving* or "product development" or national or population* or industry or ((limit* adj3 content) or (portion adj3 size*) or "Unit pricing" or "unit prices" or (Serving* adj3 size*) or (choice adj5 (food* or drink* or beverage* or health*))))	6,190,037
4	#2 AND #3	1,621,348
5	#1 OR #4	9,053,594
6	TS=((Food adj product*) or foodstuff* or foods or Nutrition or Meals or beverage* or drink* or nutrition* or nutrient* or calori* or kilocalori* or energy intake or fat* or trans-fat* or trans-fatty acid* or TFA or salt* or sodium or sugar* or "energy density" or "energy dense" or (food adj3 ingredient*) or (food adj3 composition*) or beverage* or Cola or Colas or Soda or sodas or (fizzy adj2 drink*) or (carbonated adj2 drink*) or (soft adj drink*) or "commercial baby food*" or "commercial infant food*" or "infant drink*" or "baby drink*")	5,195,188
7	TS=(reformulat* or "re-formulat*" or (formulat* adj5 (ingredient* or recipe* or processing or manufact* or novel or new)) or (recipe* adj5 (develop* or novel or new or ingredient* or composition or processing or manufact*)))	32,309
8	#5 AND #6 AND #7	1,293
9	limit to 2013	1,032

CINAHL + Econlit (EBSCO)

1	TI (polic* or legislation* or pledge* or ban or bans or law or mandatory or legal or regulat* or self-regulat* or quasi-regulation or co-regulat* or government* or guideline* or codes or "industry code" or "code or practice" or compliance or standards) OR AB (polic* or legislation* or pledge* or ban or bans or law or mandatory or legal or regulat* or self-regulat* or quasi-regulation or co-regulat* or government* or guideline* or codes or "industry code" or "code or practice" or compliance or standards)	1,477,208
2	TI (standard* or strategy or strategies or intervention* or restriction* or voluntary or public private partnership* or initiative* or program* or reformul*) OR AB (standard* or strategy or strategies or intervention* or restriction* or voluntary or public private partnership* or initiative* or program* or reformul*)	1,778,690
3	TI (label* or advertising or marketing or sales or packag* or serving* or "product development" or national or population* or industry or ((limit* adj3 content) or (portion adj3 size*) or "Unit pricing" or "unit prices" or (Serving* adj3 size*) or (choice adj5 (food* or drink* or beverage* or health*)))) OR AB (label* or advertising or marketing or sales or packag* or serving* or "product development" or national or population* or industry or ((limit* adj3 content) or (portion adj3 size*) or "Unit pricing" or "unit prices" or (Serving* adj3 size*) or (choice adj5 (food* or drink* or beverage* or health*))))	1,170,465
4	S2 AND S3	389,778
5	S1 OR S4	1,701,346
6	TI ((Food adj product*) or foodstuff* or foods or Nutrition or Meals or beverage* or drink* or nutrition* or nutrient* or calori* or kilocalori* or energy intake or fat* or trans-fat* or trans-fatty acid* or TFA or salt* or sodium or sugar* or "energy density" or "energy dense" or (food adj3 ingredient*) or (food adj3 composition*) or beverage* or Cola or Colas or Soda or sodas or (fizzy adj2 drink*) or (carbonated adj2 drink*) or (soft adj drink*) or "commercial baby food*" or "commercial infant food*" or "infant drink*" or "baby drink*") OR AB ((Food adj product*) or foodstuff* or foods or Nutrition or Meals or beverage* or drink* or nutrition* or nutrient* or calori* or kilocalori* or energy intake or fat* or trans-fat* or trans-fatty acid* or TFA or salt* or sodium or sugar* or "energy density" or "energy dense" or (food adj3 ingredient*) or (food adj3 composition*) or beverage* or Cola or Colas or Soda or sodas or (fizzy adj2 drink*) or	567,290

	(carbonated adj2 drink*) or (soft adj drink*) or "commercial baby food*" or "commercial infant food*" or "infant drink*" or "baby drink*")	
7	TI (reformulat* or "re-formulat*" or (formulat* adj5 (ingredient* or recipe* or processing or manufact* or novel or new)) or (recipe* adj5 (develop* or novel or new or ingredient* or composition or processing or manufact*))) OR AB (reformulat* or "re-formulat*" or (formulat* adj5 (ingredient* or recipe* or processing or manufact* or novel or new)) or (recipe* adj5 (develop* or novel or new or ingredient* or composition or processing or manufact*)))	3,272
8	S5 AND S6 AND S7	451
9	limit to 2013	396

Proquest – ASSIA, ABI Inform Global, PAIS

1	TI,AB(polic* or legislation* or pledge* or ban or bans or law or mandatory or legal or regulat* or self-regulat* or quasi-regulation or co-regulat* or government* or guideline* or codes or "industry code" or "code or practice" or compliance or standards)	3,110,867
2	TI,AB (standard* or strategy or strategies or intervention* or restriction* or voluntary or public private partnership* or initiative* or program* or reformul*)	30,710
3	TI,AB(label* or advertising or marketing or sales or packag* or serving* or "product development" or national or population* or industry or ((limit* adj3 content) or (portion adj3 size*) or "Unit pricing" or "unit prices" or (Serving* adj3 size*) or (choice adj5 (food* or drink* or beverage* or health*))))	3,024,634
4	2 AND 3	5,723
5	1 OR 4	3,114,558
6	TI,AB((Food adj product*) or foodstuff* or foods or Nutrition or Meals or beverage* or drink* or nutrition* or nutrient* or calori* or kilocalori* or energy intake or fat* or trans-fat* or trans-fatty acid* or TFA or salt* or sodium or sugar* or "energy density" or "energy dense" or (food adj3 ingredient*) or (food adj3 composition*) or beverage* or Cola or Colas or Soda or sodas or (fizzy adj2 drink*) or (carbonated adj2 drink*) or (soft adj drink*) or "commercial baby food*" or "commercial infant food*" or "infant drink*" or "baby drink*")	684,474
7	TI,AB(reformulat* or "re-formulat*" or (formulat* adj5 (ingredient* or recipe* or processing or manufact* or novel or new)) or (recipe* adj5 (develop* or novel or new or ingredient* or composition or processing or manufact*)))	7701
8	5 AND 6 AND 7	205
9	limit to 2013	110

Cochrane Library CENTRAL

1	(polic* or legislation* or pledge* or ban or bans or law or mandatory or legal or regulat* or self-regulat* or quasi-regulation or co-regulat* or government* or guideline* or codes or "industry code" or "code or practice" or compliance or standards):ti,ab	150588
2	MeSH descriptor: [Public Policy] this term only	91
3	MeSH descriptor: [Health Policy] this term only	351
4	MeSH descriptor: [Nutrition Policy] this term only	427
5	MeSH descriptor: [Food Labeling] this term only	248
6	MeSH descriptor: [Government Regulation] this term only	61
7	MeSH descriptor: [Legal Epidemiology] this term only	0
8	#1 OR #2 OR #3 OR #4 OR #5 OR #6 OR #7	151101
9	(standard* or strategy or strategies or intervention* or restriction* or voluntary or public private partnership* or initiative* or program* or reformul*):ti,ab	777243
10	(label* or advertising or marketing or sales or packag* or serving* or "product development" or national or population* or industry or ((limit* NEAR/3 content) or (portion NEAR/3 size*) or "Unit pricing" or "unit prices" or (Serving* NEAR/3 size*) or (choice NEAR/5 (food* or drink* or beverage* or health*)))):ti,ab	269904

11	#9 AND #10	138203
12	#8 OR #11	261291
13	((Food NEAR product*) or foodstuff* or foods or Nutrition or Meals or beverage* or drink* or nutrition* or nutrient* or calori* or kilocalori* or energy intake or fat* or trans-fat* or trans-fatty acid* or TFA or salt* or sodium or sugar* or "energy density" or "energy dense" or (food NEAR/3 ingredient*) or (food NEAR/3 composition*) or beverage* or Cola or Colas or Soda or sodas or (fizzy NEAR/2 drink*) or (carbonated NEAR/2 drink*) or (soft NEAR drink*) or "commercial baby food*" or "commercial infant food*" or "infant drink*" or "baby drink*"):ti,ab	213269
14	MeSH descriptor: [Food] explode all trees	44827
15	MeSH descriptor: [Beverages] explode all trees	8560
16	MeSH descriptor: [Infant Food] explode all trees	1711
17	#13 OR #14 OR #15 OR #16	235161
18	(reformulat* or "re-formulat*" or (formulat* NEAR/5 (ingredient* or recipe* or processing or manufact* or novel or new)) or (recipe* NEAR/5 (develop* or novel or new or ingredient* or composition or processing or manufact*))):ti,ab	5260
19	#12 AND #17 AND #18	299
20	#19 with Cochrane Library publication date Between Jan 2013 and Apr 2023	247

Grey literature

**World Cancer Research Fund NOURISHING Database**<br>Search = reformulation (NOURISHING database only)<br>85 results
**WHO institutional repository** https://apps.who.int/iris/<br>Search = (food OR drink) AND (reformulation)<br>1695<br>Sorted by relevance, screened the first 800
**World Obesity Federation** https://www.worldobesity.org/)<br>Food reformulation = 162
**NCD Alliance**<br>Tagged with nutrition - Publications & Multimedia Statements, Submissions & Briefings
**Google**<br>Incognito window to reduce biases generated by search history, and restricted to files in a PDF format only. Restricted to the first 50 results.<br>• (food OR drink OR diet OR nutri*) AND (reformulat* OR regulat*) filetype:pdf<br>24
**BASE Bielefeld Academic Search** https://www.base-search.net/ limit by report to avoid journal paper<br>(food OR drink) AND (reformulation) ….. limited to X... search results = x. 129 screened on TnA and 29 to be screened on FT
**OSF preprint**<br>(food OR drink OR diet OR nutri*) AND (reformulat* OR regulat*)<br>15 pages (10 per page) – sorted on relevance
**Policy commons**<br>https://policycommons.net/<br>Search for '(food OR drink)' AND reformulation<br>8,968 results – sorted by relevance<br>Screened the first 100
**CORDIS European Commission (europa.eu)**<br>101 results<br>'food' AND 'reformulation'

Planning stage

**Google Scholar**<br>The 'cited by' feature on Google Scholar was used to examine the citations of related systematic reviews and key articles that became known to the review team during the planning stages of the review. Sorted by relevance we would examine at minimum the first 10 pages, with restrictions on the date from 2013 onwards.

Table S3. Step-by-step guide to conducting/replicating this review

Step 1	Conduct all database searches and export the results
Step 2	Conduct all grey literature results and export the results (where possible in a format that can be imported to chosen software)
Step 3	Remove all duplicates
Step 4	Complete citation searching (now or after full-text screening)
Step 5	Screen on title and abstract in double
Step 6	Reconcile title and abstract screening with the review team
Step 7	Screen on full-text in double
Step 8	Reconcile full screening with the review team and finalise the includes
Step 9	Complete data extraction, bias and quality assessment in double

Table S4. Full descriptive table of the included studies

Author, year	Country	Design and sample	Policy details	Product reformulation details and sector	Comparison	Nutrients of interest	Key findings
**Studies with mandatory policies/interventions (n = 18)**							
Ale-Chilet, 2022	Chile	Pre-post<br><br>Monthly product-level sales; nutrition data from two stores; New Product Database	Mandatory Chilean FOPL Warning Labels and advertising restrictions for products exceeding established thresholds for sodium, sugar, fats and calories – stage 1, 2016 and stage 2, 2018	Breakfast cereal (in)	2000–2016 vs 2016–2019	Sugar<br>Calories<br>Sodium<br>SFA	Post-implementation the estimated net effect was a significant decrease of 17kcal, 1.3g sugar, 0.05g SFA and 5mg sodium per 100g of cereal purchased, compared to pre-implementation.
Barahona, 2022	Chile	Pre-post<br><br>Walmart-Chile data (all transactions between May 2015 -March 2018)	Mandatory Chilean FOPL Warning Labels and advertising restrictions - stage 1, 2016 thresholds (22.5g for sugar/ 350kcal for energy)	Breakfast cereal (in)	2015 vs 2018	Sugar<br>Calories<br>Weighted average decrease in caloric/sugar concentration	Post-implementation, 13 of 55 products reduced their concentration of calories to below the threshold and 9 of 27 products reduced their sugar content to be below the threshold. The weighted average of the caloric and sugar concentration of products decreased (significance not tested/reported).
Bates, 2020	United States	Cross-sectional<br><br>2014 Nielsen sales data; Nutrition Facts label (NFL) data; additional nutrition data collected from grocery store or retailer websites	FDA regulated FOP sodium content and nutrient content-based claims, introduced in 1993 and last updated in 2017	Sodium-modified foods and regular, matched counterparts within four food categories: soups, processed meats, vegetables, and savory snacks (in)	Nutrition content of sodium-modified foods with nutrition claims vs their regular counterparts	Sodium<br>Calories<br>Total fat<br>SFA<br>Protein<br>Carbohydrate<br>Sugar<br>Potassium	Compared with regular foods, sodium-modified foods had significantly less sodium per serving in all food categories. Similarly, compared with regular foods, sodium-modified foods had significantly less sodium per 100 mg in all food categories except soup. Compared with their regular counterparts, sodium-modified foods had no significant differences in the calories, total carbohydrate, sugar, protein, and total fat content.
Champion, 2020	France	Repeated cross-sectional<br><br>The French Observatory of Food Quality database (2008, 2011, 2018 -2020)	Mandatory BOP nutritional labeling INCO regulation for energy, fat, SFA, carbohydrates, sugars, proteins and salt (three stages of implementation: 2008, 2011, 2018)	Breakfast cereals (in)	2011 vs 2018	Fat<br>SFA<br>Sugars<br>Sodium<br>Fibre	Between 2011 and 2018, several cereal families significantly improved their nutritional content, including reduced saturated fat, salt, and sugar, along with increased fibre.
De Kock, 2016	South Africa	Repeated cross-sectional<br><br>Representative analysis samples of the stock cube powder	Mandatory regulations for the gradual reduction of salt over a period of six years (stage 1, 2016 and stage 2, 2019)	Stock cubes (in)	2013 pre-regulation vs 2016 phase 1 regulation	Sodium	The sodium level in the stock cubes (used to make chicken stews) decreased according to the guidelines, from 498 to 328 mg/100ml (significance not tested).
Garsetti, 2016	United States	Repeated cross-sectional<br><br>Nielsen sales volume data (2010-2013); private label products from seven retailers (2011); Food products from local supermarkets (2002)	2006 Mandatory BOP declaration of TFA	Top selling spreads and margarines brands (in)	Spreads sold in 2002 (arithmetic average of the sample) vs 2013 (Nielsen data)	Total fat<br>SFA<br>MUFA<br>PUFA<br>TFA<br>(sales volume weighted average)	From 2002 to 2013, the fat content of spreads reduced by 2.2g per 14g serving, with TFA decreasing by 1.5g. The fat composition improved, with a decrease in solid fat (from 39% to 30% of total-fatty acids) and an increase in unsaturated fat (from 61% to 70% of total-fatty acids). Additionally, 86% of spreads no longer contained partially hydrogenated vegetable oils (significance not tested).
Grummon, 2021	United States	Pre-post<br><br>MenuStat database on food products by the New York	2018 FDA compliance of menu calorie labelling for food establishments with 20 or more US locations, outlined in the	35,354 menu items sold at 59 large chain restaurants (OOH)	2012 vs 2019	Calories	Between 2012 to 2019 there was a significant decrease in the calorie content of newly introduced items (compared to new items introduced before labelling) but no change in

		City Department of Health and Mental Hygiene (NYC DOHMH) (2012)	2010 Patient Protection and Affordable Care Act				the calorie content of continuously offered items.
Jahn, 2018	United States	Pre-post<br><br>Data comprised photographs of all pre-packaged competitive foods and beverages; Nutrition data from the package label, manufacturer websites or online retailer	2012 Massachusetts school standards food and beverage regulations – limiting calories, portion sizes, saturated and TFA, sugar, and sodium of snack foods and beverages offered to children, and emphasizing additive-free water, skim and 1% milk, fruits and vegetables, and whole grains	Pre-packaged competitive foods and beverages (in)	2012 vs 2013 vs 2014	Sodium<br>Energy<br>Total fat<br>SFA<br>Sugars<br>Fibre<br>Protein	After 2012, energy, SFA, sodium and sugar decreased, and fibre increased significantly among all competitive foods. By 2013, 8% of foods were reformulated, as were an additional 9% by 2014.
Martinovic, 2020	France	Repeated cross-sectional<br><br>OQALI database (2010/2017)	Mandatory BOP nutritional labeling INCO regulation for energy, fat, SFA, carbohydrates, sugars, proteins and salt (three stages of implementation: 2008, 2011 & 2018)	Sauces (in)	2010 vs 2017	Fat<br>SFA<br>Sugars<br>Sodium<br>Fibre<br>Protein	On average, significant changes were observed for: bolognese and similar sauces decrease in fat, SFA, and sugar content; fish sauces decrease in sodium content; cooked tomato increase in fibre content, tomato-cheese sauces decrease in fat and protein content. There was a significant increase in the average sugar content of private label brand meat sauces.
Monge-Rojas, 2017	Latin America	Repeated cross-sectional<br><br>Food stuff data on food commonly consumed in four cities in Latin America	Mexico and Costa Rica: 2010 mandatory declaration of TFA content if there is a fat or cholesterol content claim<br><br>Brazil: 2007 prohibition of vegetable oil hydrogenation and mandatory declaration of TFA content of their pre-packaged foodstuffs<br><br>Argentina: 2014 legislation to limit industrially produced-TFA content	Selected foods containing TFA (in and OOH)	2011 vs 2015	TFA	There was a significant decrease in the content of TFA in the sampled foods across all sites and all sample products met the recommended levels of TFA content.
Quintiliano, 2020	Chile	Pre-post<br><br>Data is sourced from nutritional information declared on products in the biggest supermarket chain from 2013 and 2019	Mandatory Chilean FOPL Warning Labels and advertising restrictions for products exceeding established thresholds for sodium, sugar, fats and calories – stage 1, 2016 and stage 2, 2018	Most commonly consumed packaged foods (solid and liquid) from 16 food groups (in)	2013 vs 2019	Energy<br>Total sugars<br>SFA<br>Sodium	From 2013 to 2019, there was a significant reduction in the median: energy content for flour-based foods, confitures and similar, fats and oils and canned food; sugar content for dairy, confitures/similar and cereals; sodium content for fats and oils and spices, condiments, and sauces. There was a significant increase in sodium for dairy and meat and derivatives groups. Out of four liquid food groups, dairy and sugary beverages, showed a significant reduction in energy and total sugar. SFA did significantly change in either solid or liquid products.
Reyes, 2020	Chile	Pre-post + repeated cross-sectional	Mandatory Chilean FOPL Warning Labels and advertising restrictions for products exceeding established thresholds for sodium, sugar,	Packaged foods and beverages from 6 major supermarkets (including	2015/2016 (pre-implementation) vs 2017(post-implementation)	Energy<br>Total Sugars<br>Saturated fats<br>Sodium	Post-implementation there was a significant decrease in the number of products classified as high-in energy (breakfast cereals, savoury spreads); high-in sugar (beverages, milk drinks, breakfast cereals, sweet baked products,

		Data is sourced from nutritional information declared on products in supermarkets from 2015 to 2017	fats and calories – stage 1, 2016 = High in limits per 100g food = 350kcal of energy, 22.5 g of sugar, 6 g of SFA, 800 mg of sodium; per 100mL liquid = 100 kcal of energy, 6 g of sugar, 3 g of SFA, 100 mg of sodium	beverages, yogurt, cereal, desserts, candy, spreads, snacks, etc.) (in)			sweet spreads, savoury spreads); high-in SFA (savoury spreads); and high-in sodium (cheeses, ready to eat meals, sausages, soup). There were no changes in any nutrients for desserts and ice creams, savory baked products, yogurts, or non-sausage meat products.
Saavedra-Garcia, 2022	Peru	Pre-post<br><br>Data was sourced from nutritional information declared on products in supermarkets, small grocery store and convenience store from 2019 to 2021	Mandatory Peruvian FOPL Warning Labels – stage 1, 2019 and stage 2, 2021	29 brands were selected for beverages and 65 brands for foods (in)	2019 vs 2021	Total sugars<br>SFA<br>Sodium<br>TFA<br>NNS	Compared to the first-phase collection (2019), there was significant decrease in sugar content in beverages with subsequent increase in NNS levels by 2021. There were no significant changes in SFA, TFA or sodium in beverages. Among food, there were significant changes in SFA, TFA and NNS but not for sugar or sodium. The percentage of the beverages that would carry FOPWL decreased from 59% to 31% and food products that would carry any FOPWL label decreased from 82% to 62%, two years after implementation.
Sisti, 2023	United States	Pre-post<br><br>Data was collected and photographed at each chain restaurants at baseline/prior to enforcement (November 2015 - January 2016) and follow-up (March-April 2017)	Mandatory government regulation for menu labelling of sodium high foods enforced from June 2016 (a sodium warning icon depicting a saltshaker next to any menu item containing ≥2,300 mg sodium). Including shareable, multi-component or customizable items	Menu items in chain full-service and quick service restaurants (OOH)	2015 (pre-enforcement) vs 2017 (post-enforcement)	Sodium	There was no significant change in sodium content when comparing items offered at follow-up to those at baseline.
Tran, 2019	United States	Repeated cross-sectional<br><br>Data was sourced from MenuStat project (2017) and restaurant websites	2018 compliance of menu calorie labelling for food establishments with 20 or more US locations, outlined in the 2010 Patient Protection and Affordable Care Act	Food items sold in U.S. convenience stores and pizza restaurant chains (OOH)	2013 vs 2017	Calories	Leading up to the implementation, median calories significantly decreased in convenience stores. Appetizers and sides showed a similar significant reduction. Pizza restaurants introduced lower-calorie pizza options in 2017, but no other significant calorie changes were observed.
Wellard-Cole, 2018	Australia	Longitudinal observational<br><br>Nutritional composition data was collected from menu items available at a single time point, each year from 2009 to 2015	2012 mandatory menu energy labelling in New South Wales: to provide consumers with information that may facilitate healthier meal choices and increase consumer knowledge of the contribution of fast food to energy intake (20 or more stores across the state or 50 or more stores nationally)	Fast-food meals from the five largest chains in Australia - Hungry Jack's, KFC, McDonald's, Oporto and Red Rooster (OOH)	Pre-implementation (2009–2011) vs post-implementation (2012–2015)	Energy	There was no evidence of a change in energy per serving in menu items available before implementation.
Zancheta, 2021	Chile	Pre-post<br><br>Nutritional composition data was collected from six major supermarket chains during January and February of 2015, 2016, and 2017	Mandatory Chilean FOPL Warning Labels and advertising restrictions for products exceeding established thresholds for sodium, sugar, fats and calories – stage 1, 2016	Packaged foods and beverages (in)	2015 vs 2017	Sugars<br>NNS	Post-implementation there was a significant increase in the use of at least one NNS in beverages, dairy-based beverages, yogurts, and desserts and ice creams but no significant changes among breakfast cereals, candies and sweet confectioneries, and sweet spreads. Reduced sugar in foods and beverages was

							noted, however results were not statistically significant.
**Studies with voluntary Interventions (n = 52)**							
Arcand, 2014	Canada	Repeated cross-sectional<br><br>TFMP (Health Canada, 2005–2009), the University of Toronto Food Label Information Program (2010–2011), and the Restaurant Database (2010)	2007 Government-set TFA limits for industry and encouraged substitution of TFAs with unsaturated fats during reformulation	Packaged, restaurant and institutional foods (in and OOH)	2005–2009 vs 2010–2011<br><br>Food meeting TFA limits vs not meeting TFA limits	TFA	The TFA content of processed foods and restaurant foods decreased but the significance was not tested. TFA limits increased from 75% to 100%, with the largest improvements seen for croissants, pies, cakes, garlic spread and bread. Processed foods that met TFA limits had significantly lower TFA levels than similar products that did not meet the TFA limits.
Bablani, 2020	Australia and New Zealand	Difference-in-differences<br><br>Nutritrack 2013 to 2019 data for NZ; FoodSwitch 2014 to 2018 data for Australia	2014 Government-led Health Star Rating FOPL introduced	Nonseasonal packaged products (in)	Australia- 2014 vs 2018<br>New Zealand- 2013 vs 2019	Energy<br>Sugars<br>SFA<br>Sodium<br>Protein<br>Fibre	Products that adopted the FOPL significantly lowered the sodium content in both NZ and Australia; only products in NZ significantly lowered the sugar content; fibre content significantly increased in NZ but significantly decreased in Australia. There were no significant changes in energy density, protein or SFA content.
Bandy, 2022	United Kingdom	Repeated cross-sectional<br><br>Nutrient composition data from the websites of 4 leading UK retailers: Asda, Morrisons, Sainsbury's, and Tesco from 2015-2020	Government-led salt reduction programme in 2003: a series of progressively lower, product-specific reformulation targets for the food industry, combined with advice to consumers to reduce salt. Subsequent targets were set for 2009, 2011, 2014, and 2017	Packaged foods (in)	2015 vs 2020	Sodium	There was a small decline in the salt content of foods and total volume of salt sold between 2015 and 2020, but observed changes were not statistically significant.
Bernstein, 2020	Canada	Longitudinal observational<br><br>University of Toronto's Food Label Information Program databases (2013 and 2017)	2015 Government-led sugar-related BOP food labelling policies and sugar intake recommendations. List to be declared by 2022 – transitional period of 5 years	Prepackaged foods and beverages (in)	2013 vs 2017	Sugar<br>Energy<br>Total fat<br>SFA<br>Sodium<br>Carbohydrate<br>Fibre<br>Protein	Overall, there was a significant decrease in mean sugar level by 12.4%.<br>By product category, 5/17 significantly lower mean sugar levels and one had significantly higher levels. In the products with reduced sugar content had significantly lower energy levels, sodium, total fats and carbohydrates and no significant changes for fibre, protein or SFA.
Brants, 2017	Netherla nds	Repeated cross-sectional<br><br>Dutch Nutrient Database (NEVO) 2011; Food data provided by the Dutch Food and Consumer Product Safety Authority (2015/2016), the Dutch Bakery Association, the chain organization for oils and fats (2016) and the manufacturers	2014 Voluntary Product Composition Improvement Agreement on the maximum content of salt, SFA and calories and sugars in foods. This agreement was signed by ministry of health, welfare sports and other sectors to make consumers aware of healthier food choices	Selected packaged food (in)	2011 vs 2016	Sodium<br>Sugar<br>SFA	Since 2011, the salt content in bread, sauces, soups, canned vegetables, and chips decreased significantly (between 12 – 26%); the SFA content in biscuits significantly decreased, but significantly increased for raw meat products. There were no significant changes for sugar content.
Christoforou, 2013	Australia	Repeated cross-sectional<br><br>Data from flagship stores of two major supermarket	2007 NGO-led sodium reduction targets (Australian Division of the World Action on Salt & Health) to	Ready meal products (in)	All years between 2008 and 2011	Sodium	Between 2008 and 2011, the overall mean sodium content in Australian ready meal products had no differences. Newly introduced products had lower sodium content compared

		chains (Coles and Woolworths) and three smaller, independent retailers (2008-2011)	reduce average population salt intake by 25%<br><br>2009 Government-set targets for sodium reduction and SFA from The Food and Health Dialogue for nine priority categories of packaged foods				to discontinued products (Significant levels not reported in tables/test).
Clapp, 2018	United States	Pre-post<br><br>National Salt Reduction Initiative (NSRI) Packaged Food Database (2009, 2012 and 2014)	2009 National Salt Reduction Initiative had voluntary, category-specific sodium targets with the goal of reducing sodium in packaged and restaurant foods by 25% over 5 years	Top-selling processed foods (in)	2009 vs 2015	Sodium<br>Calories	Between 2009 and 2015, there were significant decreases in serving size, calories per serving, calorie density, sodium per serving, and sodium density in top-selling processed foods. These changes in calorie density did not correspond to changes in sodium density. Regardless of whether calorie or sodium density decreased, increased, or remained the same, there was a decline in sales.
Curtis, 2016	United States	Repeated cross-sectional<br><br>National Salt Reduction Initiative Packaged Food Database (2009, 2012 and 2014)	2009 National Salt Reduction Initiative had voluntary, category-specific sodium targets with the goal of reducing sodium in packaged and restaurant foods by 25% over 5 years	Packaged foods (in)	2009 vs 2014	Sodium	In 2009, no food categories met the National Salt Reduction Initiative 2012 or 2014 sodium reduction targets. By 2014, the sales-weighted mean sodium density declined significantly in almost half of all food categories.
Eyles, 2013	United Kingdom	Repeated cross-sectional<br><br>Household consumer panel by UK Kantar Worldpanel (2006); Nutrition Information Panels data (2006 onwards)	2006 Government-led sodium reduction targets for more than 80 categories of processed food	Processed foods (in)	2006 vs 2011	Sodium	Between 2006 and 2011, UK foods saw a significant reduction in crude sodium content, with a mean reduction of 26 mg/100 g (7%).
Eyles, 2018	New Zealand	Repeated cross-sectional<br><br>Food and beverage products data collected from >20 major fast-food chains in NZ; Nutrient data from company websites	2015 Government-led HSR FOPL and a government initiated 'The Healthy Kids Industry Pledge' where food companies have been encouraged to make voluntary pledges to improve their products for children	12 food groups and 10 major fast-food chains (OOH)	2012 vs 2016<br><br>5-year trend	Energy<br>Sodium	A moderate and significant reduction over time noted in sodium density. Increases were observed in energy density and per serve, however not statistically significant.
Fichera, 2020	UK	Difference-in-differences<br><br>Kantar Worldpanel data on grocery purchases (2005-2008)	2007 Multiple Traffic Light FOPL – solo and hybrid with Guideline Daily Amounts (before government endorsement)	Packaged food (in)	Dataset from 2005-2008 comparing pre vs post implementation specific to each retailer (Waitrose and Co-Op = 2006; Marks & Spencer and ASDA = 2007)	Nutritional quality calculated using the UK Nutrient Profiling Score (nutrients = calories, SFA, Sugar, Sodium)	Post-implementation, the nutritional quality of the labelled products significantly improved, compared to pre-implementation.
Garcia, 2020	United Kingdom and Latin America (Mexico, Ecuador,	Cross-sectional<br><br>Ofcom Nutrient Profiling System	UK government 2017 voluntary sugar reduction programme across nine specific food groups	Ready-to-eat breakfast cereals (REBCs) and yoghurts (in)	United Kingdom vs Latin America (taxation of sugar-sweetened beverages in	Sugar	Sugar content in REBCs in the UK was significantly lower than in Ecuador, Mexico, and Guatemala. Yoghurts had similar sugar content across countries.

	and Guatema la)				Mexico and Ecuador)		
Gressier, 2021	United Kingdom	Repeated cross-sectional<br><br>Dietary intakes from the National Diet and Nutrition Survey (NDNS) rolling program	UK government 2003 salt reduction program: education campaigns to raise awareness about the risks associated with a high-salt diet and of a reformulation strategy for food manufacturers	Food and beverage (in)	2008–2009 vs 2016–2017	Sodium	Sodium density in solid foods consumed reduced by 15%, primarily driven by reformulation (13% decrease) and product renewal (3% decrease) in categories like bread and meat (significance not tested).
Hashem, 2019	United Kingdom	Repeated cross-sectional<br><br>Data on sugar (1992) was obtained from a booklet by Octavo and 2017 data was collected from product packaging of the major London supermarket chains	2016 Government-led sugar reformulation programme with target of reducing sugar by 20% by 2020 across nine food and drink categories	Chocolate confectionery (in)	1992 vs 2017	Sugar	The average sugar content across chocolate products was significantly higher in 2017 compared to 1992.
He, 2014	United Kingdom	Repeated cross-sectional<br><br>Data collected from food companies and surveys	Voluntary salt reduction programme created by Consensus Action on Salt and Health and Government-led collaboration with the food industry. Salt targets set in 2006 for industry to reach by 2010	Processed food (in)	2001 vs 2011 (comparison of all the years between)	Sodium	There was a reduction in salt content in all food categories (significance not tested and comparison year varies between products).
Health Canada, 2018	Canada	Pre-post<br><br>Sales data and nutrition fact tables (2017)	Government-established a Sodium Working Group to develop a strategy for reducing sodium intake in 2007	Processed foods (in)	2012 vs 2016	Sodium	The reduction of sodium in processed foods was much lower than anticipated. For most food categories, manufacturers did not do enough to reduce the sodium levels in the foods that they sell to Canadians (significance not tested).
Jensen, 2017	Denmark	Longitudinal observational<br><br>Sales and reformulation data from a Denmark food retail chain (2013-2014)	Retailer-led product reformulation 'silent' strategy (reformulation without claims) in 2013/2014	Retailer-brand food products (mayonnaise, fruit yogurt, breads, muesli) (in)	March 2013 vs February 2014	Calories	There was an average reduction in calorie content for all product categories analysed (between 4-18%)(significance not tested).
Kanter, 2019	Chile	Pre-post<br><br>Data comprised photographs of products from six supermarkets entered into an electronic data management platform REDCap (2015-2016)	Anticipatory reformulation before mandatory Chilean FOPL Warning Labels and advertising restrictions for products exceeding established thresholds for sodium, sugar, fats and calories – stage 1, 2016	Packaged food and beverages from supermarket chains<br>(in)	2015 vs 2016	Sodium<br>Calories<br>Sugar<br>SFA	No product category experienced a change of more than 5% on average for energy or any nutrient content (significance not tested). Less than 2% of products would have avoided at least one warning label with reformulation.
Levi, 2018	Australia	Repeated cross-sectional George Institute (TGI) Branded Food Composition Database; Nutrient data were from TGI Branded Food Composition database (2011-2014)	2009 Government-set targets for sodium reduction from The Food and Health Dialogue (FHD) for nine priority categories of packaged foods	Soup products (in)	2011 vs 2014<br><br>FHD participants vs non-participants	Sodium	Significant reductions in sodium were observed for FHD participants but not for non-participants. Mean sodium levels significantly decreased between 2011 and 2014. The majority of FHD products met national sodium reduction targets.
Lowery, 2020	Colombi a	Pre-post	2016 "Responsible self-regulation" agreement signed by beverage industry - promising to	Packaged food and beverages (in)	2016 vs 2018	Total fat<br>SFA<br>TFA	There were significant reductions in the median quantities of calories, total sugar and free sugar contents in beverages with proportionate

		Nutrition Facts Panel (NFP) data from 5 Columbian supermarket chains and package data captured by Research Electronic Data Capture	limit advertising and sales of unhealthy beverages in schools and to provide smaller portion sizes and low-calorie beverage options, among other commitments			Sodium<br>Total sugar<br>Free sugar<br>Energy density<br>NNS	increase in NNS between 2016 and 2018. No significant change was seen for any nutrients in packaged foods.
Luger, 2018	Austria	Repeated cross-sectional<br><br>Survey of all beverages available in large Austrian supermarket outlets	NGO led 'beverage checklist' from 2010 with voluntary targets to decrease free sugars added to beverages with ≤7.4 g free sugars/100 ml and no artificial or natural intense sweeteners (guiding criteria) (Special Institute for Preventive Cardiology and Nutrition scientific association)	Sugar-sweetened beverages<br>(in and OOH)	2010 vs 2017<br><br>Beverages not fulfilling the guiding criteria vs those fulfilling them	Free sugar	There was a significant reduction of sugar content by 3.5 % between 2010 and 2017. The reduction remained significant for both groups (i.e., beverages not fulfilling the guiding criteria and those fulfilling them (≤7.4 g free sugars/100 ml and no artificial sweeteners).
McMenemy, 2022	Ireland	Repeated cross-sectional<br><br>Kantar Worldpanel market data from six major retailers (2014-2017)	Industry-led reformulation efforts to align the population's diet with the European Food Safety Authority food-based dietary guidelines	Cereals, bread, spreads, milks (in)	2014 vs 2017	Energy<br>Protein<br>Total fat<br>SFA<br>Sodium<br>Total sugar<br>Carbohydrate<br>Fibre<br>Vitamin D/B12<br>Calcium<br>Iron	Between 2014 and 2017, the were no significant changes in the composition per 100g of cereals, bread, spreads, and milks. The changes in nutrients by more than 5% were: cereal (increased = fibre; decreased = SFA, sugar, sodium); breads (increased = SFA; decreased = fibre, sodium); spreads (increased = carbohydrates, Vitamin D; decreased = sugars); milks (increased = SFA; decreased = fibre, salt, Vitamin D).
Ni Mhurchu, 2017	New Zealand	Repeated cross-sectional<br><br>Survey of packaged food labelling and composition in supermarkets; Nutrition Information Panel (NIP) data (2014-2016)	2014 Government-created HSR FOPL	Packaged food and beverage (in)	2014 vs 2016<br><br>HSR vs non-HSR products	Energy<br>SFA<br>Sugars<br>Sodium<br>Protein<br>Fibre	There were significant changes in mean energy, sodium, and fiber between 2014 and 2016; but changes were not significant for other nutrients. Products displaying HSR FOPLs had significantly lower mean SFA, total sugar and sodium contents, and higher fibre content, compared to non-HSR labelled products.
Moore, 2020	United Kingdom	Repeated cross-sectional<br><br>Survey of yogurts from 5 UK based supermarkets	2017 Government-led sugar reformulation programme to reduce sugar content of products that contribute the most sugar to children's intakes (including yogurt) by 20% by 2020, including an initial 5% reduction in the first year (compared to sugar levels in the foods in 2015)	Yogurt products (in)	2016 vs 2019	Total sugar	There was a significant reduction in the median total sugar contents between 2016 and 2019.
Moran, 2022	United States	Repeated cross-sectional<br><br>NSRI Packaged Food Database (2009-2018)	2009 Government-created National Salt Reduction Initiative (a national partnership of health organizations) with sodium reduction targets for packaged and restaurant foods by 20 percent over 5 years: 10% reduction in sodium by 2012 and 25% reduction by 2014 in 62 categories of packaged foods	Packaged foods (in)	2009 vs 2012 vs 2014 vs 2018	Sodium	There was a significant decrease in mean sodium levels between all subsequent study years from 2009 to 2018. There was a significant increase in the proportion of US packaged foods meeting the 2012 targets and 2014 targets between 2009 and 2012 with no further improvements through subsequent years.

Morrison, 2019	Australia	Repeated cross-sectional<br><br>Data on products from three large regional supermarkets in South-East Queensland; nutrition information panel data	2014 Government-created Health Star Rating FOPL	Children's packaged product (in)	2013 vs 2016<br><br>HSR labelled vs non-HSR labelled	Energy<br>Saturated fat<br>Total sugar<br>Sodium<br>Protein<br>Fibre	For products available in both 2013 and 2016, the mean sodium content significantly decreased but there was no significant change in mean energy content. By product category, there was a significant decrease in sugar and SFA and significant increase in protein and fibre for cereal products there was no significant change in the nutrient composition for dairy or fruit-based products.
Nilson, 2017	Brazil	Repeated cross-sectional<br><br>Data is sourced from mandatory food label information of products, from the official company websites or customer services	Sodium reduction targets set by the Ministry of Health, Pan American Health Organization, the Brazilian Food Industry Association to gradually reduce in the maximum sodium content of packaged foods through biannual targets for the food categories that most contribute to sodium intake	Processed foods (in)	2011 vs 2013	Sodium	Between 2011 and 2012/2013 there were significant reductions in the sodium content of instant pastas, breads, cakes, cake mixes, cake rolls, mayonnaise, sweet and salted crackers, and condiments.
Nilson, 2017	Brazil	Repeated cross-sectional<br><br>Data is sourced from mandatory food label information of products, from the official company websites or customer services	Sodium reduction targets set by the Ministry of Health, Pan American Health Organization, the Brazilian Food Industry Association to gradually reduce in the maximum sodium content of packaged foods through biannual targets for the food categories that most contribute to sodium intake	Processed Foods (in)	2011 vs 2017	Sodium	Between 2011 and 2017 there were significant reductions in the sodium content of bread, cake mixes, cakes, instant pastas, mayonnaise, dairy spreads, margarines, mozzarella cheese, bouillon cubes and powders, sweet biscuits, filled cookies and salted crackers.
Ning, 2017	New Zealand	Repeated cross-sectional<br><br>Sales volume and sodium levels data from 13 companies; market data from A.C. Nielsen New Zealand	NGO-led Tick endorsement FOPL introduced in 1991 (National Heart Foundation of New Zealand)	Processed foods (breakfast cereals, edible spreads, processed poultry and cooking sauces) (in)	2011 vs 2013	Sodium	There was a large reduction of salt in all products tested, equivalent to 16 tonnes removed (but significance was not tested). Through both the reformulation and formulation of new products.
Park, 2020	South Korea	Repeated cross-sectional<br><br>Data sourced from 2010-14 Korea National Health and Nutrition Examination Survey	2012 Government-led sodium reduction plan – target to reduce sodium intake by 20%; five key components including voluntary reformulation of processed foods to lower sodium content	Processed food sources rich in sodium (in and OOH)	2010 vs 2013	Sodium	The sodium content of kimchi, soy sauce, soybean paste, red pepper pastes and instant noodles decreased (by 13-40%), significance not tested. Results were adapted from the Korean article Kim, 2015 (English translation was unavailable)
Pérez-Farinós, 2016	Spain	Repeated cross-sectional<br><br>Data sourced from nutrient information panels from 2010 and 2015	2011 Government-set Food Safety and Nutrition Act to reduce artificial TFA in food products	Food products acquired from hyper/supermarkets countrywide (in)	2010 vs 2015	TFA<br>TFA/Total fat	There were significant decreases in TFA content and TFA/total ratio for 5/16 product categories (salty snacks, confectionary and pastries, chocolates, biscuits and chips) and no significant changes in the rest of the product categories.
Pérez-Farinós, 2018	Spain	Repeated cross-sectional<br><br>Sample of bread loaves collected in bakeries and supermarkets from all	A 2004 collaboration agreement between the Spanish Agency for Food Safety and Nutrition (regulatory agency of the Spanish Government), the Spanish Confederation of Bakers and the	Bread (in and OOH)	2008 vs 2014	Sodium	There was no significant change in salt content between 2008 and 2014, indicating that the amount of salt in common Spanish bread remained stable over that period.

		autonomous cities and communities in Spain (2014)	Spanish Association of Manufacturers of Frozen Dough to reduce salt content of bread by 25% within four years to reach an average amount of 1.63 g/100 g product				
Pinho-Gomes, 2023	Australia	Repeated cross-sectional<br><br>Data is sourced from George Institute's FoodSwitch Monitoring Datasets (2015, 2016, 2017, 2018 and 2019)	2018 Industry-led 'Sugar Reduction Pledge' which retroactively applied from 2015 (Australian Beverages Council) to reduce sugar by 20 percent over the Pledge period (2015-2025)	Non-alcoholic beverage (in)	2015 vs 2019	Sugars	No evidence that the voluntary sugar reduction pledge had led to a significant change in the sugar content of beverages available for purchase in Australia.
Pombo-Rodrigues, 2017	United Kingdom	Repeated cross-sectional<br><br>Data is sourced from product packaging and nutrient information panels from 2004 (salt only), 2006, 2009, 2012 and 2015	2003 Government and NGO created salt reduction programme (Consensus Action on Salt and Health)	Breakfast cereals (in)	2004 vs 2015 for salt<br><br>1992 vs 2015 for sugar	Sodium<br>Free sugars	There was significant reduction in salt level by 47% in cereals between 2004 and 2015. Sugars content reduced by 12% between 1992 and 2015 (statistically insignificant).
Pravst, 2017	Slovenia	Repeated cross-sectional<br><br>Data is sourced from nutrient composition of prepacked foods in supermarkets, mega markets and a discount market from 2011 and 2015	2010 Government National Action Plan: cutting the population's salt intake to the recommended 5g daily before 2020	Prepacked foods from major food store (in)	2011 vs 2015	Sodium	The average sodium content decreased, but not significantly.
Russell, 2021	Australia	Repeated cross-sectional<br><br>Data sourced from Mintel Global New Product database	Government-created Health Star Rating FOPL	All new Australian food and beverage products displaying an HSR (in)	2014 vs 2020	Added sugar<br>NNS<br>Overall healthfulness (assessed by HSR)	The proportion of new products sweetened with added sugars and the number of new products displaying a HSR increased over time, while NNS use did not.
Savio, 2013	Australia	Pre-post<br><br>Data is sourced from Nutrient Information Panels on products in a large supermarket (2009 and 2011)	NGO-led Tick endorsement FOPL (Heart Foundation) created in 1989<br><br>2007 NGO-led sodium reduction targets (Australian Division of the World Action on Salt & Health)<br><br>2009 Government-set targets for sodium and SFA reduction from The Food and Health Dialogue	Food and beverage products marketed to children (in)	2009 vs 2011	Energy<br>Protein<br>Total fat<br>SFA<br>Sugar<br>Fibre<br>Sodium<br>Healthiness	Mixed results, with similar levels of positive and negative reformulation across the nutrients (positive = ≥10% decrease in energy, total fat, SFA, sugars or sodium content or ≥10% increase in fibre content and negative = reformulation the other way). More products were positively reformulated for SFA and sodium content (significance not tested).
Sparks, 2018	Australia	Repeated cross-sectional<br><br>Data is sourced from Australian FoodSwitch database (2010, 2013, 2015 and 2017)	2009 Government-set targets for sodium and SFA from Food and Health Dialogue and Healthy Food Partnership Targets in nine priority food categories	Processed meat (in)	2010 vs 2017<br><br>Products with the presence of a sodium target vs absence of a sodium target<br><br>Food and Health Dialogue targets vs Healthy Food	Sodium	Between 2010 and 2017, the median sodium content significantly reduced for processed meat, bacon, meat with pastry pancetta and prosciutto, sausages, sliced meat, but significantly increased for canned meat. There was statistically significant reduction in the median sodium level of processed meats with targets while there was no change in processed meats without a target. The Healthy Food Partnership had a significantly lower proportion of products at or below the reduction target

					Partnership Targets		compared to Food and Health Dialogue in 2010 and 2017.
Spiteri, 2018	France	Repeated cross-sectional<br><br>Data sourced from French Food Quality Observatory database and Kantar Worldpanel database	Government-set food reformulation and collective agreements between 2008-2011 from industry to improve the nutritional quality of products including SFA, sodium, sugar (public-private National Food Program)	Grocery basket products (in)	2008 vs 2013	Sugar<br>Fats<br>SFA<br>Fibre<br>Sodium	There was a decrease in the sales-weighted average sodium content, sugar content and SFA content.
Spiteri, 2018	Australia	Repeated cross-sectional<br><br>Data sourced from Mintel Global New Products database, AUSNUT food composition database (2011–13), company websites and Calorie King	2012 Industry-led Healthier Australia Commitments to improve the nutritional quality of their product portfolios by reducing sodium by 25%, SFA by 25% and energy by 12.5% (Australian Food and Grocery Council)	Food and beverages (in)	Companies committed vs not committed	Energy<br>Protein<br>SFA<br>Carbohydrate s Sugar<br>Fibre<br>Sodium	Companies who voluntarily committed launched a significantly greater proportion of unhealthy foods (classified as red/HFSS, discretionary or ultra-processed) and significantly fewer healthy foods (classified as green, core foods and minimally processed). Specific nutritional composition not reported.
Tan, 2019	United Kingdom and China	Repeated cross-sectional<br><br>Data sourced from nutrition information panels on products sold in grocery retail stores in China (2015-2017) and UK (2008-2018)	2003 NGO and government-led Salt Reduction Programme (Consensus Action on Salt and Health), with voluntary maximum salt targets set for industry to reach by 2017	Sauces (in)	2008 vs 2018<br><br>UK (reformulation plans) vs China (no reformulation plan/strategy)	Sodium	In the UK the median salt content decreased significantly in 8/17 product categories. The median salt content of UK products was significantly lower than Chinese products. 70% of UK products met the UK 2017 maximum salt targets.
Tassy, 2022	Global	Repeated cross-sectional<br><br>Mintel Global New Products database and Nutrient profiling system	WHO nutritional recommendations set in 2013 (Global Action Plan for the Prevention and Control of NCDs 2013–2020) on fat, sugar and sodium	Packaged food (in)	Products released pre-2016 vs products released post 2016 (up until 2020)	Total sugars<br>Sodium<br>SFA<br>Protein<br>Healthfulness (assessed by Health star rating)	Products released after 2016 were significantly lower in sugar and sodium than before 2016; however, no clear trend was observed for SFA, protein, or fibre.
Theis, 2019	United Kingdom	Cross-sectional<br><br>Data sourced from restaurant websites	Government-led 2011 Public Health Responsibility Deal which companies signed up to, encouraging restaurants to provide menu labelling at the point of purchase for energy and nutrition (including sodium)	Menu items served by UK chain restaurants (OOH)	Restaurants with voluntary menu labelling vs without	Energy<br>Fat<br>SFA<br>Carbohydrate s<br>Sugar<br>Protein<br>Sodium	Menu items served with voluntary menu labelling had significantly less fat and less salt than those from restaurants without menu labelling. There were no significant changes for energy, SFA, carbohydrates, sugar or protein.
Thomson, 2016	New Zealand	Repeated cross-sectional<br><br>Data sourced from National Heart Foundation database, supermarket sales data purchased from Nielsen Ltd and by approaching product manufacturers	NGO-led Tick endorsement FOPL introduced in 1991 (National Heart Foundation of New Zealand)	Tick and non-Tick packaged products (in)	2011 vs 2013<br><br>Tick vs non-tick products	Energy<br>SFA<br>TFA<br>Sodium<br>Fibre<br>Calcium	Between 2011 and 2013, the nutritional content of Tick products was reformulated to be lower in energy, SFA, calcium and sodium and higher in fibre (significance not tested/reported). Tick labelled products were lower in energy, SFA, TFA and sodium than similar non-Tick products in the food category (significance not tested/reported).
Trevena, 2014	Australia	Repeated cross-sectional<br><br>Data sourced from mandatory Nutrition	2009 Government established Food and Health Dialogue targeting sodium reduction in	Breads, breakfast cereals and processed meats (in)	2010 vs 2013<br><br>Companies publicly	Sodium	Between 2010 and 2013 there was a significant reduction in the sodium level in bread, breakfast cereal and processed meat products. The percentage of products meeting

		Information Panel (NIP) on product packaging from four Australian supermarkets	food products – set in 2010 to be met end of 2013		committed to the targets vs non participants		the targets also increased significantly (ranging from 28-42% before to 47-67% after). There was no observable difference between the companies committed to meeting the targets and not.
Trevena, 2015	Australia	Repeated cross-sectional<br><br>Data sourced from mandatory Nutrition Information Panel (NIP) on product packaging	2009 Government established Food and Health Dialogue targeting sodium reduction in food products – set in 2010 to be met end of 2013	Mixed packaged food stocked in supermarkets (in)	2011 vs 2013<br><br>Supermarket private-label vs branded foods	Sodium	Between 2011 and 2013 there was a small but significant reduction in the overall mean sodium reduction for both supermarket and branded products (≤2%). Supermarket products, including new products, had significantly lower mean sodium content than branded products.
Van Dam, 2022	France	Cross-sectional<br><br>Data sourced from Open Food Facts,<br>Mintel Global New Products or brand websites	2017 Government-created Nutri-Score FOPL<br><br>2007 Government created industry pledges – 'Voluntary Commitment Charter for Nutritional Progress' to reduce levels of salt, sugar, TFA and fat	Packaged food, non-alcoholic beverages, and food at quick-service restaurants (in and OOH)	Voluntary nutrient company commitments	Overall product portfolio healthiness (assessed using Nutri-Score and NOVA classification)	There was no relationship observed between voluntary company commitments and healthier product portfolios.
Van Der Bend, 2020	Netherlands	Repeated cross-sectional<br><br>Data sourced from the Dutch Choices Foundation using the Dutch Choices Logo between 2006 and 2012	Dutch Choices logo endorsement FOPL created in 2006 - NGO-led (with industry and Government involvement). Applied to packaged foods or on signs in catering settings to products deemed as healthy	Basic (products that contribute significantly to daily essential nutrients intake) and non-basic food products with Choices Logo criteria (in)	'Old' (2006/2007, 2007/ 2008 or 2008/2009) vs 'New' (2015/2016)<br><br>Basic product vs non-basic products	Energy<br>Sodium<br>SFA<br>TFA<br>Added sugar<br>Fibre	The energy density, SFA, TFA, added sugar and sodium of all 'New' FOP-labelled products (both basic and non-basic) was significantly lower than all of the 'Old' labelled products; fibre content was significantly higher in New' FOP-labelled products.
Vergeer, 2022	Canada	Repeated cross-sectional<br><br>Nutritional information sourced from University of Toronto Food Label Information Program 2013 and 2017 database	Industry led reformulations: governments have encouraged industries to voluntarily include sodium reduction in their guidelines, policies and procedures. The Food Company Reformulation tool (FCR) quantifies the strength of voluntary reported recent actions and commitments made by food companies with scores calculated out of 100%	Packaged food and beverage (in)	2013 vs 2017<br><br>Company commitments vs actual reformulation (Higher FCR score vs lower FCR score)	Calories<br>Sodium<br>SFA<br>TFA<br>Free sugar<br><br>Total sugar<br><br>Overall healthfulness (Health Star Rating)	Between 2013 and 2017, calories, sodium, TFA and free sugars significantly decreased; the overall healthiness and SFA content significantly increased; and no changes were observed for total sugar. The association between FCR scores and overall healthiness were negative (higher FCR = lower HSR) and positively associated with sugars (higher FCR = higher sugar content). There was no relationship observed between higher FCR score and product reformulation relating to calories, sodium, SFA or TFA.
Vermote, 2020	Belgium	Repeated cross-sectional<br><br>Nutritional composition data was sourced for breakfast cereals available in seven different supermarket chains (2017-2019)	Government-led Nutri-Score FOPL	Breakfast cereals (in)	2017 vs 2018	Energy<br>Sodium<br>SFA<br>Fat<br>Carbohydrate<br>Total sugar<br>Fibre<br>Protein	A significant reformulation of cereals was seen with a decrease in sugar, carbohydrates, and sodium; and an increase in fibre and protein. There were no statistical changes for SFA or energy content.
Vlassopoulos, 2017	United States	Repeated cross-sectional	Nestlé Nutritional Profiling System: A guide for the formulation or reformulation of	Nestlé products from eight packaged food	2009–2010 vs 2014–2015	Energy<br>Sodium<br>Total fat	The percentage of products meeting all the nutrient targets for the category (classified as YES) increased between 2009–2010 to 2014–

	and France	Data sourced from Nestlé Nutritional Profiling System (NNPS)	foods and beverages created in 2004, but updated with the last update in 2014 – applied between 2009-10 and 2014-15	categories (USA = pizza, milk-based beverages, water ice and sorbet, and complete meals; France = children's ice cream, centre of plate food items, soups, cold sauces) (in)	Products meeting nutrient targets (YES) vs products not meeting the targets (NO)	SFA<br>Total sugar	2015. Overall, for most products there a decrease in SFA and total fat (≥10 %), energy (<10 %), sodium (up to 22%) and in total sugars (up to 31%) (significance not tested/reported).
Yon, 2014	United States	Pre-post<br><br>Nutrition profiles for flavored milk before and after reformulation were provided by the school nutrition director or the school's milk supplier	Anticipatory reformulation of milk products before updated Government set school standards came into effect 2012 –milk offerings limited to fat-free or low fat (1%) and must be fat free if flavored, and limits on calories	Milk and fat-free flavored milk (in)	Before reformulation (between 2008-2009) vs after reformulation (2009-2010)	Added sugar<br>Calories<br>Fat<br>Total sugars	After reformulation, the flavored milks contained fewer calories and fat (significance not tested).
Zupanič, 2019	Slovenia	Repeated cross-sectional<br><br>Nutritional composition data was sourced from major grocery chains in 2015 and repeated in 2017	2015 Government-led voluntary industrial commitments (public-private) to reduce free sugar content of food and beverages	Packaged foods and non-alcoholic beverages (in)	2015 vs 2017	Free sugar	The results were mixed, with some product categories decreasing (chocolate, breakfast cereals) and some increasing (meal replacements, yogurts) in mean free sugar content (significance not tested).
**Studies with both mandatory and voluntary interventions (n = 7)**							
Hooker, 2014	United States and Canada	Repeated cross-sectional<br><br>Mintel/GNPD (Global New Products Database) data (2006-12)	Mandatory: inclusion of TFA content on BOP NFT (Dec 2005 Canada/ Jan 2006 US)<br><br>Mandatory: 2006 US FOP nutrient claim threshold for 'trans fat free' if less than 0.5g/serving<br><br>Mandatory: Canada FOP nutrient claim threshold for 'trans fat free' if less than 0.2g/serving<br><br>Voluntary: Government-set recommendations in Canada to reduce TFA set in 2005 to be met by 2009 1) limit TFA content of oils/spreads to 2% of total fat content 2) limit TFA content for all other foods to 5% of total fat content	Cookies (in)	Nutrient quality of new cookies launched between 2006-12 in Canada and the US	TFA	The content of TFA in cookies in both countries significantly decreased
Moz-Christofoletti, 2021	22 European countries	Repeated cross-sectional<br><br>Market share data, sales data and nutrition composition data provided by Euromonitor International (2005-2019)	Mandatory: 2012 EU directive for the prohibition of added sugars in fruit juices and the 2019 EU regulation on TFA content 2g/100g of fat<br><br>Voluntary: EU wide Frameworks for National Salt Initiatives (2008), National Initiatives on	Packaged food and beverages – nine categories for each nutrient (in)	2015 vs 2018	Sugar<br>Sodium<br>SFA<br>Fibre	The weighted mean differences for the following nutrients significantly decreased (SFA for processed meat and biscuits; sugar for soft drinks; salt for processed meat; fibre for ready meals) and significantly increased for sugar in biscuits and salt in baked goods. None of the nutrients were significantly different in the rest of the products

			selected nutrients (2011), Annexes on Saturated Fat (2012) and Added Sugars (2015) and the roadmap for Action on Food Product Improvement (2016)				
Otite, 2013	United States	Longitudinal observational Data is sourced from Nutrition Fact panels on products sold in supermarkets (2007, 2008, 2010, and 2011)	Mandatory: 2006 US FOP nutrient claim threshold for 'trans fat free' if less than 0.5g/serving<br><br>Voluntary: supermarket retailer-led reformulation of TFA	US supermarket food products (in)	2007 vs 2008 vs 2010 vs 2011	TFA	The mean content of TFA per serving significantly decreased between each of the comparison years
Temme, 2017	Netherlands	Repeated cross-sectional<br><br>Data sourced from the Dutch Food Composition database, Innova database, Food Label database and chemical analytical data by the Dutch Food Safety Authority (NVWA) and Dutch Bakery Association	Mandatory: Commodities Act in effect from 2013 for maximum level of salt in bread (not more than 1.8% calculated on the dry matter)<br><br>Voluntary: 2007 industry-created Taskforce Salt Reduction to reduce salt in processed foods by 12% in 2010	Major processed foods contributors to dietary salt intake and bread (In)	2011 vs 2016	Sodium	The average salt content of bread, sauces (tomato meat and ketchup), canned vegetables and soups were significantly lower in 2016 compared to 2011. The salt levels for cheese and processed meats were lower, but the differences were not significant
Urban, 2014	United States	Longitudinal observational Data sourced from archival website of three fast-food chain restaurants	Mandatory: restrictions on partially hydrogenated fat use in some use cities – including New York City in 2006<br><br>Voluntary: reformulation to meet Government set 2010 Dietary Guidelines for Americans recommendations – to reduce added sugars, solid fats, and sodium	French fries, cheese burgers and grilled chicken sandwich from three fast-food chains (OOH)	2000 vs 2012	Sodium<br>SFA<br>TFA	Overall, there was a significant decrease in TFA and SFA content for fries, but not for burgers; no significant decreases in sodium content for any of the products
Wellard-Cole, 2019	Australia	Repeated cross-sectional<br><br>An audit of nutritional information was conducted for Australian fast-food chains in 2016	Mandatory: 2012 menu energy labelling in New South Wales, 2015 Australian Capital Territory and 2012 South Australia state legislation requiring retail food outlets (with more than 20 outlets in the state/ 50 nationally) to display nutrition information on menus at point-of-sale<br><br>Voluntary industry-led: Quick Service Restaurant Industry Initiative for Responsible Advertising and Marketing to Children that aims 'to ensure that only food and beverages that represent healthier choices are promoted directly to children	Fast-food children's meals (OOH)	Pre-legislation period 2010 vs post-legislation 2016<br><br>QSRI signatory chains vs non-signatory chains	Sugar<br>SFA<br>Energy<br>Sodium	Between 2010 and 2016, the overall energy, sodium, SFA and sugar content of meals per serving available changed very little (significance not tested/reported). Between 2010 and 2016, there were no significant differences in proportion of meals that exceeded either 30 % or 100 % of children's recommendations for energy or any nutrient (results reported in text, not in table). The proportion of meals from QSRI signatory chains exceeded 30 % and 100 % of daily recommendations and exceeded the QSRI nutrient criteria for energy (significance not tested/reported)
Zganiacz, 2017	Australia	Repeated cross-sectional	Mandatory: BOP nutrition information panels on nearly all packaged foods (2013)	130 processed foods (in)	1980 vs 2013<br>1995 vs 2013	Sodium	There was a significant overall decrease in sodium content of processed foods (by 23% since 1980, and 12% since 1995). By product

		Nutritional composition data was collected from Australian supermarkets in 2013	Voluntary NGO-led: Tick endorsement FOPL (Heart Foundation) created in 1989<br><br>Voluntary NGO-led: 2007 sodium reduction targets (Australian Division of the World Action on Salt & Health)<br><br>Voluntary government-set targets: 2009 for sodium reduction and SFA from The Food and Health Dialogue				category, significant decreases in sodium were observed for convenience and snack foods, but no significant differences for processed meats, bakery products or cheese. Approximately 62% of the analysed products met Australian reformulation targets

FOPL, front of pack label; TFA, trans-fatty acids; SFA, saturated fatty acids; MUFA, mono-unsaturated fatty acids; PUFA, poly-unsaturated fatty acids, NNS, Non-nutritive sweeteners; NGO, non-governmental organisations; WHO, World Health Organization; BOP, back of pack; OQALI, French Observatory of Food Quality

Table S5. Sensitivity analysis for the vote counting

**Removal of the very low quality studies**
40 total<br>37 with a positive direction of effect, 3 with a negative direction of effect<br>Proportion favouring reformulation = 37/40 = 93%<br>Confidence interval = 0.8014, 0.9742<br>P-value = =2*BINOM.DIST(3, 37, 0.5, TRUE) = 0.00000012

Figure S1. Harvest plots summarising the direction of the reformulation influence by implementation of the non-fiscal policies or interventions A) mandatory B) voluntary C) both. The size of the bar indicates the study quality: largest = moderate quality; middle = low quality; smallest = very low quality.

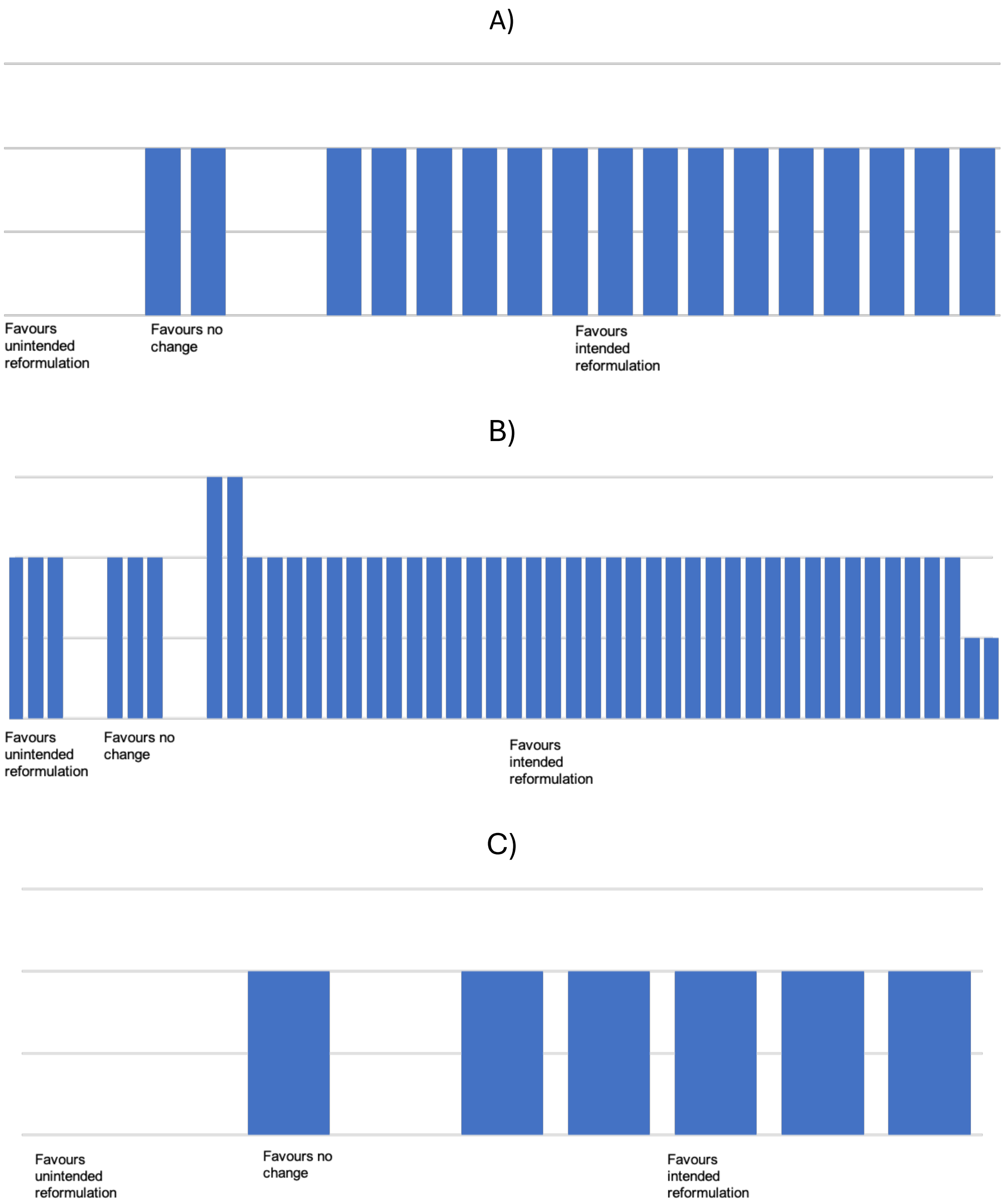

Table S6. Overall bias assessment for each of the included studies

Author	Bias assessment	Author	Bias assessment
Arcand	Low concerns	Nilson	Low concerns
Ale-Chilet	Low concerns	Ning	Minor concerns
Bablani	Low concerns	Otite	Low concerns
Bandy	Low concerns	Park	Low concerns
Barahona	Low concerns	Pérez-Farinós, 2016	Low concerns
Bates	Low concerns	Pérez-Farinós, 2018	Minor concerns
Bernstein	Low concerns	Pinho-Gomes	Low concerns
Brants	Minor concerns	Pombo-Rodrigues	Low concerns
Champion	Minor concerns	Pravst	Low concerns
Christoforou	Minor concerns	Quintiliano	Minor concerns
Clapp	Low concerns	Reyes	Minor concerns
Curtis	Low concerns	Russell	Low concerns
De Kock	Low concerns	Saavedra-Garcia	Minor concerns
Eyles, 2013	Low concerns	Savio	Low concerns
Fichera	Low concerns	Sisti	Minor concerns
Garcia	Low concerns	Sparks	Low concerns
Garsetti	Minor concerns	Spiteri (Australia)	Low concerns
Gressier	Low concerns	Spiteri (France)	Low concerns
Grummon	Low concerns	Tan	Minor concerns
Hashem	Low concerns	Tassy	High-level concerns
He	High-level concerns	Temme	Low concerns
Health Canada	High-level concerns	Theis	Low concerns
Hooker	Low concerns	Thomson	Minor concerns
Jahn	Low concerns	Tran	Low concerns
Jensen	Minor concerns	Trevena, 2014	Low concerns
Kanter, 2019	Minor concerns	Trevena, 2015	Low concerns
Levi	Low concerns	Urban	Low concerns
Lowery	Minor concerns	Van Dam	Low concerns
Luger	Low concerns	Van Der Bend	Minor concerns
Martinovic	Minor concerns	Vergeer, 2022	Low concerns
McMenemy	Low concerns	Vermote	Low concerns
Monge-Rojas	Low concerns	Vlassopoulos	High-level concerns
Moore	Low concerns	Wellard-Cole, 2018	Low concerns
Moran	Low concerns	Wellard-Cole, 2019	Minor concerns
Morrison	Minor concerns	Yon	Low concerns
Moz-Christofoletti	Low concerns	Zancheta	Low concerns
Ni Mhurchu	Low concerns	Zganiacz	Low concerns
Nilson	Low concerns	Zupanič	Low concerns
